# Residual hepatocellular carcinoma after oxaliplatin treatment has increased metastatic potential in a nude mouse model and is attenuated by Songyou Yin

**DOI:** 10.1186/1471-2407-10-219

**Published:** 2010-05-20

**Authors:** Wei Xiong, Zheng-Gang Ren, Shuang-Jian Qiu, Hui-Chuan Sun, Lu Wang, Bin-Bin Liu, Qi-Song Li, Wei Zhang, Xiao-Dong Zhu, Liang Liu, Wen-Quan Wang, Zhao-You Tang

**Affiliations:** 1 Liver Cancer Institute, Zhongshan Hospital, Fudan University, Shanghai 200032, China

## Abstract

**Background:**

The opposite effects of chemotherapy, which enhance the malignancy of treated cancers such as hepatocellular carcinoma (HCC), are not well understood. We investigated this phenomenon and corresponding mechanisms to develop a novel approach for improving chemotherapy efficacy in HCC.

**Methods:**

Human hepatocellular carcinoma cell lines HepG2 (with low metastatic potential) and MHCC97L (with moderate metastatic potential) were used for the in vitro study. An orthotopic nude mouse model of human HCC was developed using MHCC97L cells. We then assessed the metastatic potential of surviving tumor cells after in vitro and in vivo oxaliplatin treatment. The molecular changes in surviving tumor cells were evaluated by western blot, immunofluorescence, and immunohistochemistry. The Chinese herbal extract Songyou Yin (composed of five herbs) was investigated in vivo to explore its effect on the metastatic potential of oxaliplatin-treated cancer cells.

**Results:**

MHCC97L and HepG2 cells surviving oxaliplatin treatment showed enhanced migration and invasion in vitro. Residual HCC after in vivo oxaliplatin treatment demonstrated significantly increased metastasis to the lung (10/12 vs. 3/12) when re-inoculated into the livers of new recipient nude mice. Molecular changes consistent with epithelial-mesenchymal transition (EMT) were observed in oxaliplatin-treated tumor tissues and verified by in vitro experiments. The Chinese herbal extract Songyou Yin (4.2 and 8.4 g/kg) attenuated EMT and inhibited the enhanced metastatic potential of residual HCC in nude mice (6/15 vs. 13/15 and 3/15 vs. 13/15, respectively).

**Conclusions:**

The surviving HCC after oxaliplatin treatment underwent EMT and demonstrated increased metastatic potential. Attenuation of EMT by Songyou Yin may improve the efficacy of chemotherapy in HCC.

## Background

Hepatocellular carcinoma (HCC) is one of the most common malignancies in many countries, including China. Radical treatment, such as surgical resection and orthotopic liver transplantation, is feasible only for patients with small tumors [1]. For the majority of patients with unresectable HCC, the response rate of systemic chemotherapy with Adriamycin, 5-fluorouracil and platinum-based combinations was reported to be approximately 20%, and no survival benefit was observed [2].

Biological deterioration after chemotherapy has recently been reported. An "opposite effect" of chemotherapy has been demonstrated in which cyclophosphamide pretreatment induced metastasis of fibrosarcoma cells in a nude mouse model [3]. Other studies also reported that in vitro exposure to chemotherapeutic agents enhanced metastatic potential in colorectal, pancreatic, breast, and ovarian carcinoma cells [4-7]. Two explanations have been proposed for this opposite effect. Chemotherapy may damage the vascular endothelium and inhibit the host antitumor system, enabling increased tumor cell extravasation and survival [3,8]. Alternatively, chemotherapy may induce epithelial-mesenchymal transition (EMT) and enhance the metastatic potential of tumor cells [4-7]. EMT is characterized by the loss of epithelial marker E-cadherin and expression of mesenchymal markers such as N-cadherin and vimentin; it is regarded as a critical step in tumor invasion and metastasis [9]. Although these studies established a relationship among in vitro chemotherapy, EMT, and enhanced tumor metastasis, the altered metastatic potential of residual cancer after in vivo chemotherapy is not completely understood.

To exclude the influence of tumor burden, vascular injury, and inhibition of host antitumor system by chemotherapy, we designed a re-inoculation experiment in a nude mouse model of human HCC to determine whether chemotherapy promotes a more malignant phenotype of residual HCC. Because platinum-based combined chemotherapy targets HCC [10,11], oxaliplatin was selected as the chemotherapeutic agent in this study. We investigated potential mechanisms, as well as the Chinese herbal extract Songyou Yin as a potential intervention.

## Methods

### Cell lines

In the present study, we used two human HCC cell lines: MHCC97L cells, which originated from MHCC97 [12,13] (established at the Liver Cancer Institute, Zhongshan Hospital, Fudan University, Shanghai, China), and HepG2 cells (American Type Culture Collection). MHCC97L cells show a moderate metastatic potential, and HepG2 cells show a low metastatic potential. All cells were cultured with Dulbecco's Modified Eagle Medium (DMEM) containing 10% fetal bovine serum (FBS) and incubated in a humidified incubator at 37°C in 5% CO_2_.

### Compounds and antibodies

Oxaliplatin was purchased from Sigma Chemical Co. (St. Louis, MO, USA). Monoclonal antibodies used in immunofluorescence, immunoblotting, and immunohistochemistry included: rabbit anti-human vimentin, Twist, and Slug (Santa Cruz Biotechnology, Santa Cruz, CA, USA), rabbit anti-human E-cadherin and Snail (Abcam Ltd., Cambridge, UK), mouse anti-human N-cadherin (Abcam Ltd, Cambridge, UK.), mouse anti-human GAPDH (Chemicon, CA, USA) and mouse anti-human Ki-67 (Dako, Glostrup, Denmark).

### Treatment of tumor cells with oxaliplatin and analysis of cell morphology

MHCC97L or HepG2 cells were plated in six-well tissue culture plates (1 × 10^5 ^cells/well). After 24 h, the medium was replaced with DMEM containing 10% FBS and 2 μmol/L oxaliplatin. After 48 h, the medium was changed, and drug treatment was terminated. Cells were allowed to recover, and when the surviving populations reached 80% confluence, cells were passaged and exposed to oxaliplatin again for 48 h. During 6 weeks of treatment this process was repeated for a total of four 48-h exposures to oxaliplatin. Cells surviving treatment (Oxa cells) were designated MHCC97L-Oxa and HepG2-Oxa, respectively. The morphological characteristics of parental and Oxa cells were compared by microscopy (Olympus, Tokyo, Japan).

### Cell migration and invasion assays

Cell migration was assessed by transwell assay (Boyden chambers) (Corning, Cambridge, MA, UK). Briefly, 6 × 10^4 ^cells in serum-free DMEM were seeded on a membrane (8.0-μm pore size) inserted in a well of a 24-well plate. DMEM containing 10% FBS was added to the lower chamber of each well. After 48 h, cells that had reached the underside of the membrane were stained with Giemsa (Sigma Chemical Co.) and counted at × 200 magnification. The cell invasion assay was carried out similarly, except that 10 μL of matrigel (BD Biosciences) was added to each well 6 h before cells were seeded on the membrane.

### Cell proliferation assay

MHCC97L and HepG2 parental and Oxa cells were incubated in 96-well plates (5 × 10^3 ^cells/well) for 24, 48, or 72 h. Cell proliferation was then determined with an MTT kit (Beyotime, Shanghai, China). Results were expressed as the absorbance of each well at 570 nm (OD570).

### Western blot analysis and immunofluorescence

The concentration of protein extracted from Oxa and parental cells was determined with the BCA Protein Assay Kit (Beyotime, Shanghai, China). The expression of E-cadherin, N-cadherin, vimentin, Snail, Slug, and Twist was determined by immunoblotting as previously described [14].

E-cadherin, N-cadherin, and vimentin expression in parental and Oxa cells were also demonstrated by immunofluorescence. Cells were grown on glass cover slips to 40%-50% confluence, and then fixed, permeabilized, and blocked. Cells were then incubated with primary monoclonal antibodies against E-cadherin, N-cadherin, and vimentin overnight at 4°C. The next day, slides were washed and incubated with anti-mouse and/or anti-rabbit fluorescein isothiocyanate (FITC)- and/or tetramethyl rhodamine isothiocyanate (TRITC)-conjugated secondary antibody (Invitrogen). Cells were counterstained with 4'-6-diamidino-2-phenylindole (DAPI) to visualize cell nuclei and visualized by fluorescence microscopy (Olympus, Tokyo, Japan).

### Animals

Male BALB ⁄ c nu ⁄ nu mice (age, 4-6 weeks old; weight, approximately 20 g) were obtained from the Shanghai Institute of Materia Medica (Chinese Academy of Science) and maintained under standard pathogen-free conditions. The experimental protocol was approved by the Shanghai Medical Experimental Animal Care Commission.

### Pilot study for animal model

To evaluate changes in metastatic potential of residual HCC cells after in vivo oxaliplatin treatment, residual cancer from the livers of oxaliplatin-treated and untreated nude mice were re-inoculated orthotopically into new recipient nude mice. Because the treated and untreated tumors needed to be histologically similar, a pilot study compared the histology of treated and untreated tumors at different time points after chemotherapy to determine the appropriate time points for re-inoculation.

A metastatic model of human HCC in nude mice using MHCC97L cells was employed for this pilot study [12]. Briefly, MHCC97L cells (5 × 10^6^) were injected subcutaneously into the upper left flank region of nude mice. When the subcutaneous tumor reached approximately 1 cm in length (approximately 4 weeks after injection), it was removed, minced into small pieces of equal volume (2 × 2 × 2 mm^3^), and transplanted into the livers of 60 different nude mice. Based on the literature [15] and the results of our previous studies, a dosage for oxaliplatin of 10 mg/kg, once a week was adopted in the present study. Oxaliplatin was administered intraperitoneally (i.p.) to randomly selected mice (oxaliplatin treatment group; *n *= 30) on days 12, 19, and 26 after inoculation; the control group (*n *= 30) received 0.2 mL of 0.9% sodium chloride (i.p.) on those days. On days 1, 2, 5, 7 and 14 after the final treatment, six mice from each group were sacrificed by cervical dislocation. The necrosis and apoptosis of tumors in each group were compared.

### Analysis of tumor necrosis and apoptosis

Paraffin-embedded sections were prepared for hematoxylin and eosin staining. Necrosis of tumor tissue was determined by comparing the surface of necrotic areas to that of the whole tumor [16]. Apoptosis was determined using a terminal transferase dUTP nick end labeling (TUNEL) assay kit (KeyGen, Nanjing, China) according to the manufacturer's protocol. The apoptosis rate was expressed as a ratio of apoptotic cells to total tumor cells.

### Re-inoculation experiment

Analysis of tumor histology in the pilot study showed that tumor necrosis and apoptosis on days 5, 7 and 14 after the final oxaliplatin treatment were not significantly different between the control group and the treated group. We therefore used day 7 tumors for re-inoculation. Orthotopic models of human HCC were established in 36 nude mice and treated with oxaliplatin (*n *= 18) or sodium chloride (*n *= 18) using the same protocol described for the pilot study. On day 7 after the final treatment, all mice were sacrificed by cervical dislocation. Tumor fragments of equal volume (2 × 2 × 2 mm^3^) from each mouse of the oxaliplatin-treated and untreated control groups were re-inoculated into the livers of each new recipient mice correspondently. The remaining tumor tissues of both groups were fixed in 10% buffered formalin and embedded in paraffin wax for histological study.

These mice, segregated into control (bearing untreated tumors, *n *= 18) and trial ( bearing oxaliplatin pre-treated tumors, *n *= 18) groups, were then kept under standard conditions. On day 42 after re-inoculation, 12 randomly selected mice from each group were sacrificed, and tumor growth and pulmonary metastasis were assessed. Tumor tissues from re-inoculated mice were also prepared for histological study. The remaining six mice from each group were kept for survival analysis.

### Characterization of the Chinese herbal extract Songyou Yin

The water-soluble Chinese herbal medicine Songyou Yin (SYY), authorized by the Chinese State Food and Drug Administration (Grant No. G20070160), includes five Chinese medicinal herbal extracts in the following proportions (w/w): *Salvia miltiorrhiza *Bge., 14.3%; *Astragalus membranaceus *Bge., 14.3%; *Lycium barbarum *L., 23.8%; *Crataegus pinnatifida *Bge., 23.8% and *Trionyx sinensis *Wiegmann, 23.8% (all from China). High-performance liquid chromatography (HPLC) fingerprinting of Songyou Yin and its five characteristic components (see additional file 1) was carried out by the Shanghai Institute of Materia Medica (SIMM), Chinese Academy of Sciences (CAS), China [17]. The SYY used in this study was produced by the Caitong Detang Chinese Traditional Medicine Pharmaceutical Factory (batch number: 090401; Shanghai, China).

### Songyou Yin treatment in the re-inoculation model beaing oxaliplatin pre-treated tumors

The orthotopic nude mice model bearing oxaliplatin pre-treated MHCC97L xenografts was established in 84 mice using the previously described protocol. Three days after re-inoculation, mice were randomized into control (*n *= 21) and SYY-treated groups (*n *= 63). The 63 nude mice in the SYY-treated group received SYY at three different doses (2.1, 4.2, and 8.4 g/kg; each dose, *n *= 21) by oral gavage once per day. Mice in the control group received 0.2 mL of 0.9% sodium chloride via oral gavage at the same time. Treatment continued for 6 consecutive weeks. On day 42 after initiation of treatment, mice from the control group and each SYY dose group (each group, *n *= 15) were sacrificed to determine tumor volume. The tumor tissue and lungs were collected for histological analysis and a pulmonary metastasis assay. The remaining mice were maintained and used for survival analysis.

### Tumor volume, lung metastasis and survival

Tumor volume was calculated as: (a × b^2^)/2, where "a" is the widest diameter and "b" is the smallest [18]. Lung metastasis was determined by examining serial sections of every lung tissue block by microscopy. Survival time was defined as the interval between the day of inoculation and the day of death.

### Immunohistochemistry

Tumor tissue was fixed, embedded, and sliced into 5-μm thick sections. Immunostaining of E-cadherin, N-cadherin, vimentin, Snail, Slug, and Twist was carried out using a standard protocol [19].

### Statistical analysis

In vitro cell migration, invasion, and proliferation assays were analyzed by Student's t-test. Necrosis and apoptosis of tumors from the animal model were also compared by Student's t-test. Tumor volume was compared by analysis of variance (ANOVA), the lung metastasis assay was analyzed using Fisher's exact test, and survival was compared with Kaplan-Meier method with a log-rank test. Statistical analysis was performed with SPSS 15.0 for Windows (SPSS Inc. Chicago, IL, USA). P < 0.05 was considered statistically significant.

## Results

### Tumor cells that survived oxaliplatin treatment showed higher motility and invasiveness and slower proliferation in vitro

**Figure 1:**
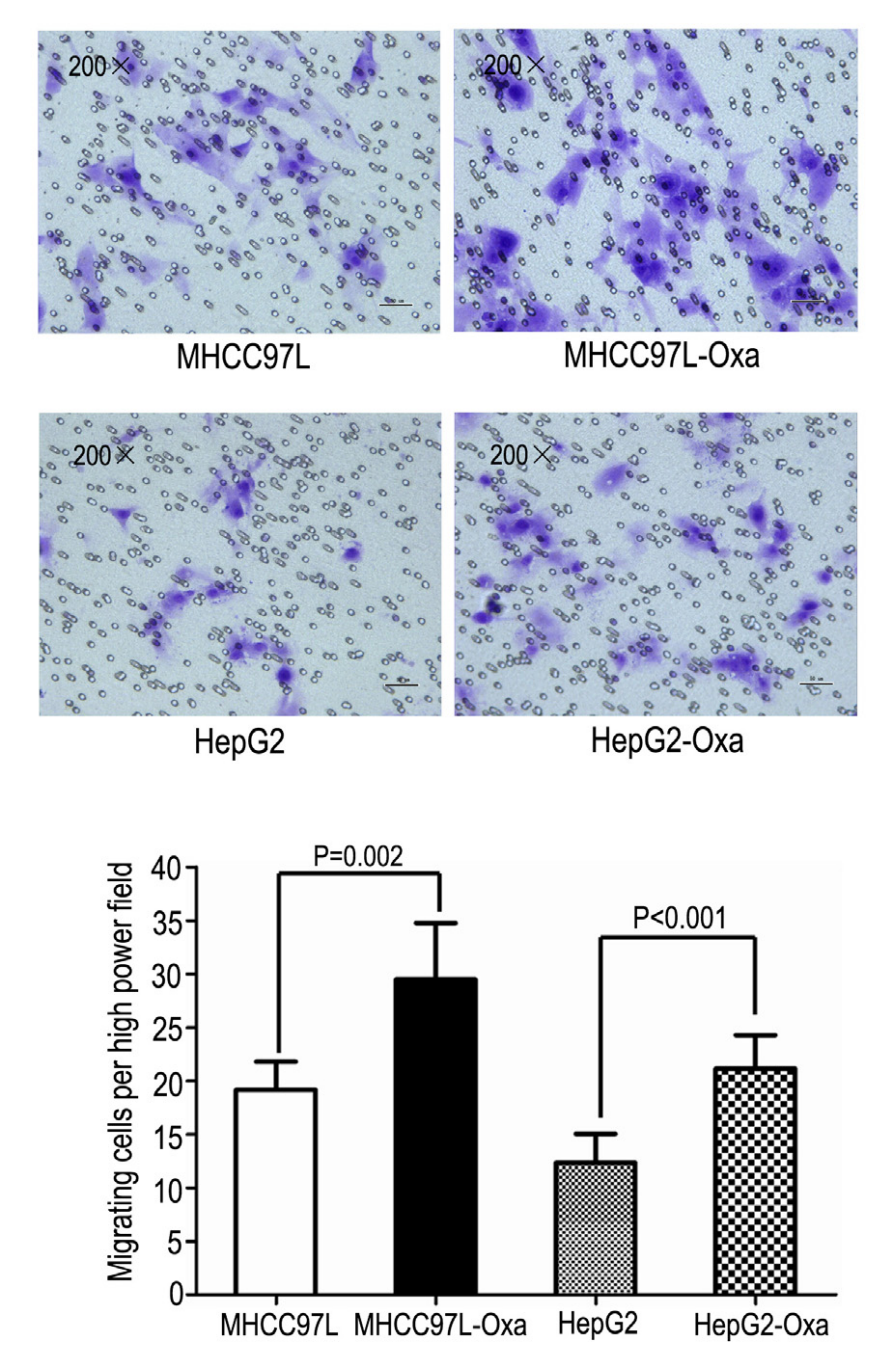
**Tumor cells that survived oxaliplatin treatment showed enhanced migration in vitro**. The transwell assay demonstrated that MHCC97L-Oxa and HepG2-Oxa cells migrated through the collagen membrane in greater numbers compared with parental MHCC97L and HepG2 cells.

**Figure 2:**
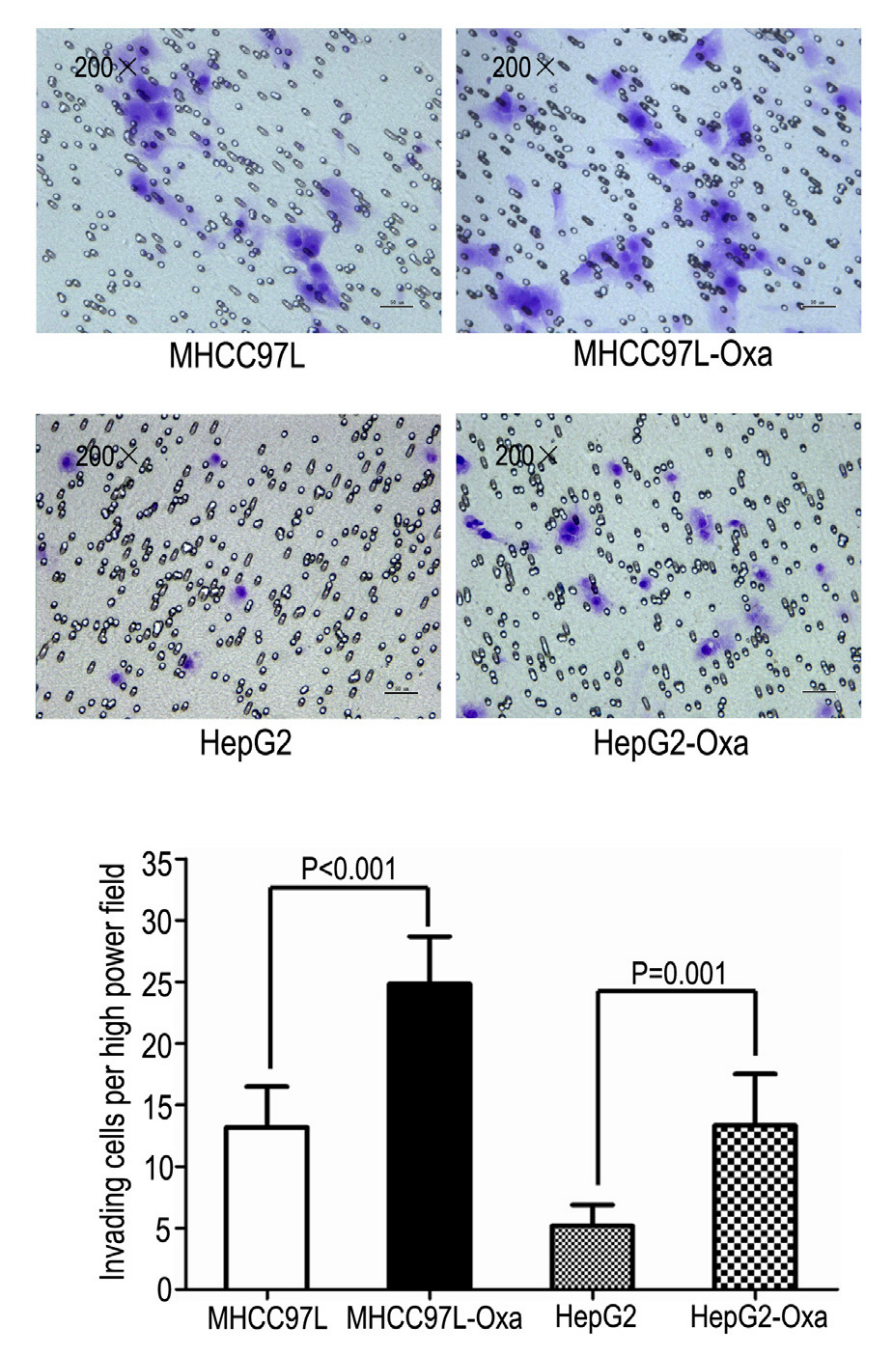
**Tumor cells that survived oxaliplatin treatment exhibited enhanced invasive properties in vitro**. MHCC97L-Oxa and HepG2-Oxa cells showed significant increase in the numbers of cells invading the Matrigel-coated membrane compared with parental MHCC97L and HepG2 cells in the modified transwell assay.

**Figure 3:**
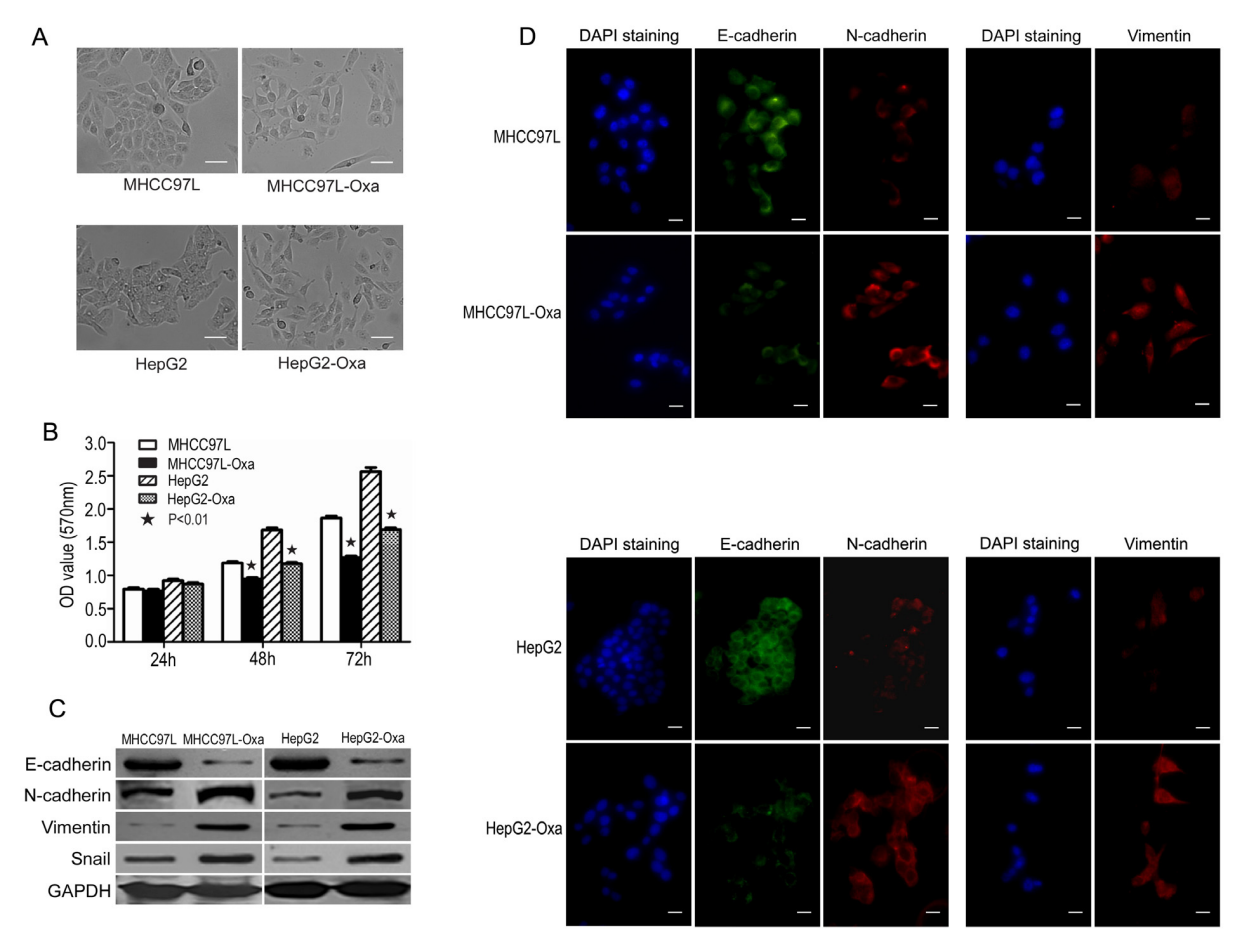
**Exposure to oxaliplatin in vitro induced epithelial-mesenchymal transition (EMT) in MHCC97L and HepG2 cells**. A) Tumor cells surviving oxaliplatin treatment were morphologically distinct from the typical epithelial appearance of parental MHCC97L and HepG2 cells, demonstrating a spindle shape with less cell-cell adhesion and increased formation of pseudopodia. B) MTT assay revealed a reduced proliferation in MHCC97L-Oxa and HepG2-Oxa cells compared with the respective parental cells. C) Western blot analysis showed molecular changes consistent with EMT in MHCC97L-Oxa and HepG2-Oxa cells (e.g., downregulation of E-cadherin and upregulation of N-cadherin, vimentin, and transcription factor Snail). GAPDH was used as an internal control. D) Reduced E-cadherin expression and increased expression of N-cadherin, and vimentin in MHCC97L-Oxa and HepG2-Oxa cells were also demonstrated by immunofluorescence.

In the migration assay (Figure 1), cells that survived oxaliplatin treatment migrated through the membrane in greater numbers compared with corresponding parental cells (MHCC97L-Oxa, 29.50 ± 5.28 vs. MHCC97L, 19.17 ± 2.64; *P=*0.002; and HepG2-Oxa, 21.17 ± 3.13 vs. HepG2, 12.33 ± 2.73; *P *< 0.001). In the invasion assay (Figure 2), MHCC97L-Oxa and HepG2-Oxa cells showed a 0.89-fold(*P *< 0.001) and 1.58-fold (*P *=0.001) increase, respectively, in the number of cells invading the matrigel-coated membrane, compared with untreated controls. At 48 and 72 h after plating, both MHCC97L-Oxa and HepG2-Oxa cells exhibited reduced proliferation compared with corresponding parental cells (Figure 3B).

### Tumor cells surviving oxaliplatin treatment exhibited EMT in vitro

MHCC97L-Oxa and HepG2-Oxa cells were morphologically distinct from the typical epithelial appearance of parental MHCC97L and HepG2 cells, demonstrating a spindle shape with less cell-cell adhesion and increased formation of pseudopodia (Figure 3A). Western blot analysis (Figure 3C) showed a significant reduction in E-cadherin expression with an upregulation of N-cadherin and vimentin in MHCC97L-Oxa and HepG2-Oxa cells. The transition from a dominant E-cadherin distribution in the cell membrane before chemotherapy to predominant N-cadherin expression after chemotherapy, known as the "cadherin switch" [20], was also demonstrated by immunofluorescence in both cell lines (Figure 3D). Furthermore, an upregulation of the transcription factor Snail was also detected in MHCC97L-Oxa and HepG2-Oxa cells (Figure 3C). However, expression of the transcription factors Twist and Slug was very low and was not affected by oxaliplatin treatment in either cell line (data not shown).

### Oxaliplatin treatment resulted in transient histological changes in tumor tissues primarily via apoptosis induction

**Figure 4:**
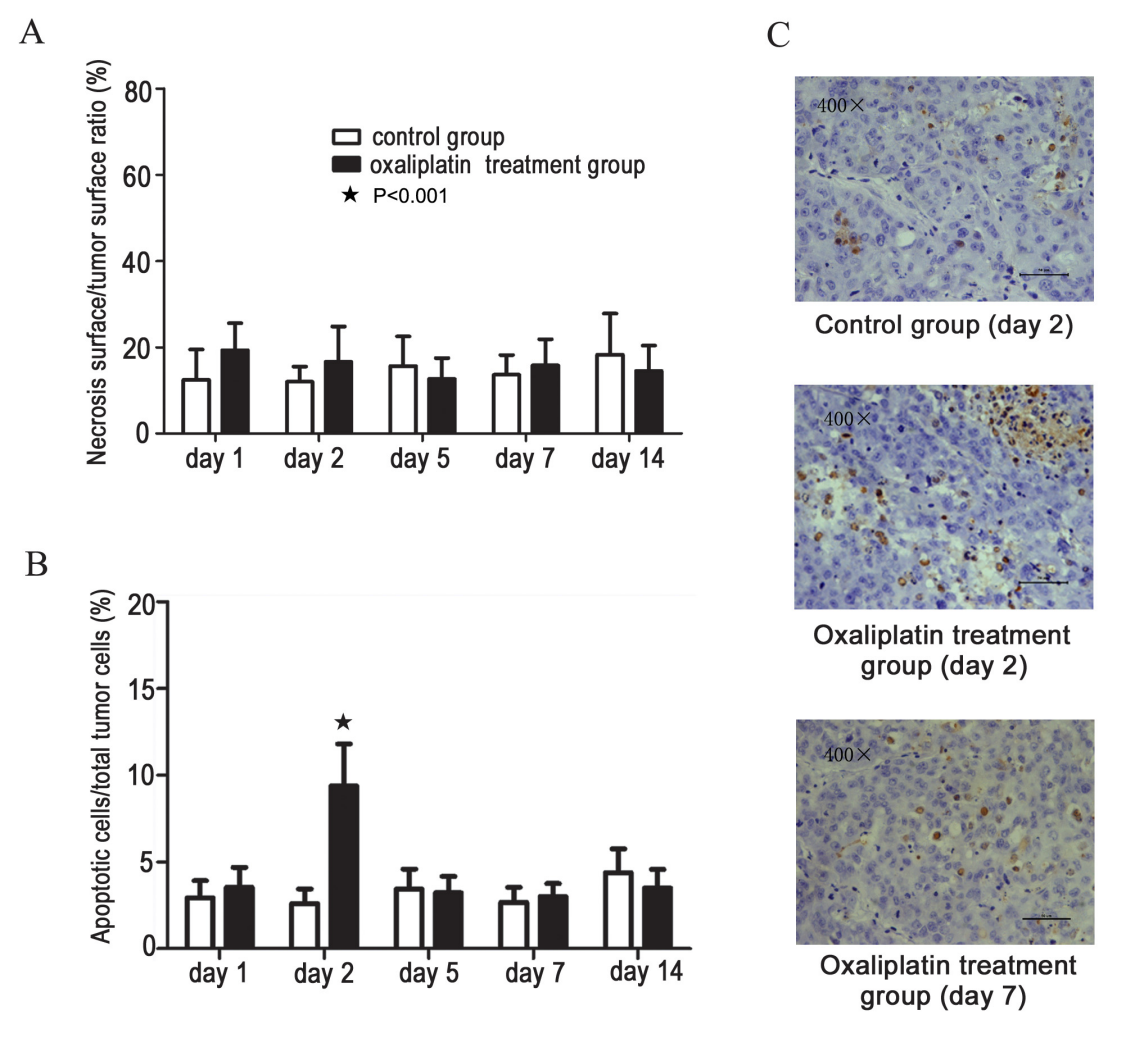
**Analysis of tumor histology after oxaliplatin treatment**. A) No significant difference in tumor necrosis was observed between the control group and oxaliplatin treatment group after treatment. B) Terminal transferase dUTP nick end labeling (TUNEL) analysis showed a basal level of apoptosis rate of about 3% in tumors of the control group throughout the 2-week experiment. In the oxaliplatin treatment group, significant apoptosis was observed on day 2 after the final injection (B, C). No significant difference in apoptosis was found between the two groups at other time points.

To explore the feasibility of re-inoculation, the histology of tumor tissues between the control and oxaliplatin treatment group was compared at various time points. As shown in Figure 4A, no significant difference in tumor necrosis was observed between the control group and oxaliplatin treatment group. Tumors in the control group maintained a basal apoptosis rate of approximately 3% throughout the 2-week experiment; however, oxaliplatin-treated tumors demonstrated significantly increased apoptosis on day 2 after the final oxaliplatin injection (Figure 4B, C). No significant difference in apoptosis was observed between the two groups at other time points.

### Residual tumors after oxaliplatin treatment exhibited enhanced metastatic potential in re-inoculated nude mice

To evaluate biological features of HCC tumors after chemotherapy, MHCC97L xenografts from the oxaliplatin-treated and untreated control groups were re-inoculated into the livers of new recipient mice. Six weeks after re-inoculation, tumor volumes in the trial (bearing oxaliplatin pre-treated tumors) and control (bearing untreated tumors) groups were 2624.59 ± 491.60 and 3849.72 ± 827.09 mm^3 ^*(P *< 0.001) respectively. In addition, Ki-67 positive cells were significantly reduced in oxaliplatin pre-treated tumors (see additional file 2).

Analysis of serial lung sections revealed that oxaliplatin pre-treated tumors gave rise to spontaneous pulmonary metastasis in 83% (10/12) of the mice in the trial group (see additional file 3). In contrast, pulmonary metastasis was found in only 25% (3/12) of the mice in the control group (*P *= 0.012).

However, no significant difference was found between the trial group and control group in mean survival time (55.17 ± 5.53 vs. 51.33 ± 4.63 days; *P *= 0.128).

### Residual tumors after chemotherapy exhibited changes consistent with EMT in nude mice

**Figure 5:**
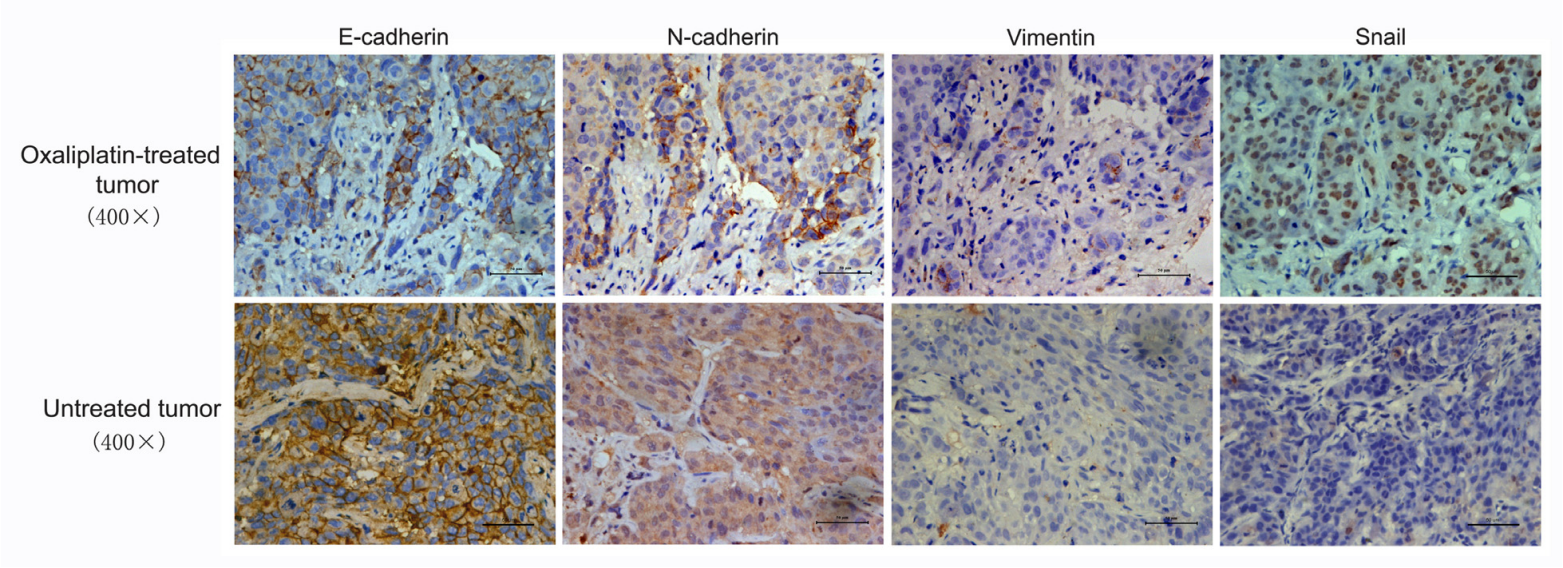
**Oxaliplatin-treated tumors exhibited changes consistent with epithelial-mesenchymal transition (EMT)**. Immunohistochemistry revealed typical membranous E-cadherin expression in the cell-cell contacts of untreated tumors. In contrast, oxaliplatin-treated tumors showed an overall reduction of E-cadherin expression, especially at the invasive front where the expression of E-cadherin was faint or even undetectable. Instead, N-cadherin was significantly upregulated in oxaliplatin-treated tumors. N-cadherin-positive tumor cells were concentrated in areas of tumor-stroma interaction and were scattered in the stroma. Increased expression of vimentin and Snail were also detected in the invasive front of oxaliplatin-treated tumors.

On day 7 after chemotherapy, oxaliplatin-treated and untreated tumors were subjected to immunohistochemistry (Figure 5). Untreated tumors showed typical membranous E-cadherin expression in the cell-cell contacts. In contrast, oxaliplatin-treated tumors showed an overall reduction of E-cadherin expression, especially at the invasive front where the expression of E-cadherin was faint or even undetectable. The mesenchymal marker N-cadherin was significantly upregulated in oxaliplatin-treated tumors. N-cadherin-positive tumor cells were concentrated primarily in areas of tumor-stroma interaction and were scattered in the stroma. Increased cytoplasmic expression of vimentin, another mesenchymal marker, was also detected in oxaliplatin-treated tumors. We also analyzed the expression of transcription factors Snail, Slug, and Twist in tumor tissues. However, only Snail was detected; it was significantly upregulated in oxaliplatin-treated tumors.

A comparison was also carried out between the control group (re-inoculated with untreated tumor) and trial group (re-inoculated with oxaliplatin pre-treated tumor), producing similar results. A much more frequent and active EMT was observed in tumors of the trial group than the control group (see additional file 4).

### Songyou Yin inhibited metastasis of oxaliplatin pre-treated tumors and increased survival

**Figure 6:**
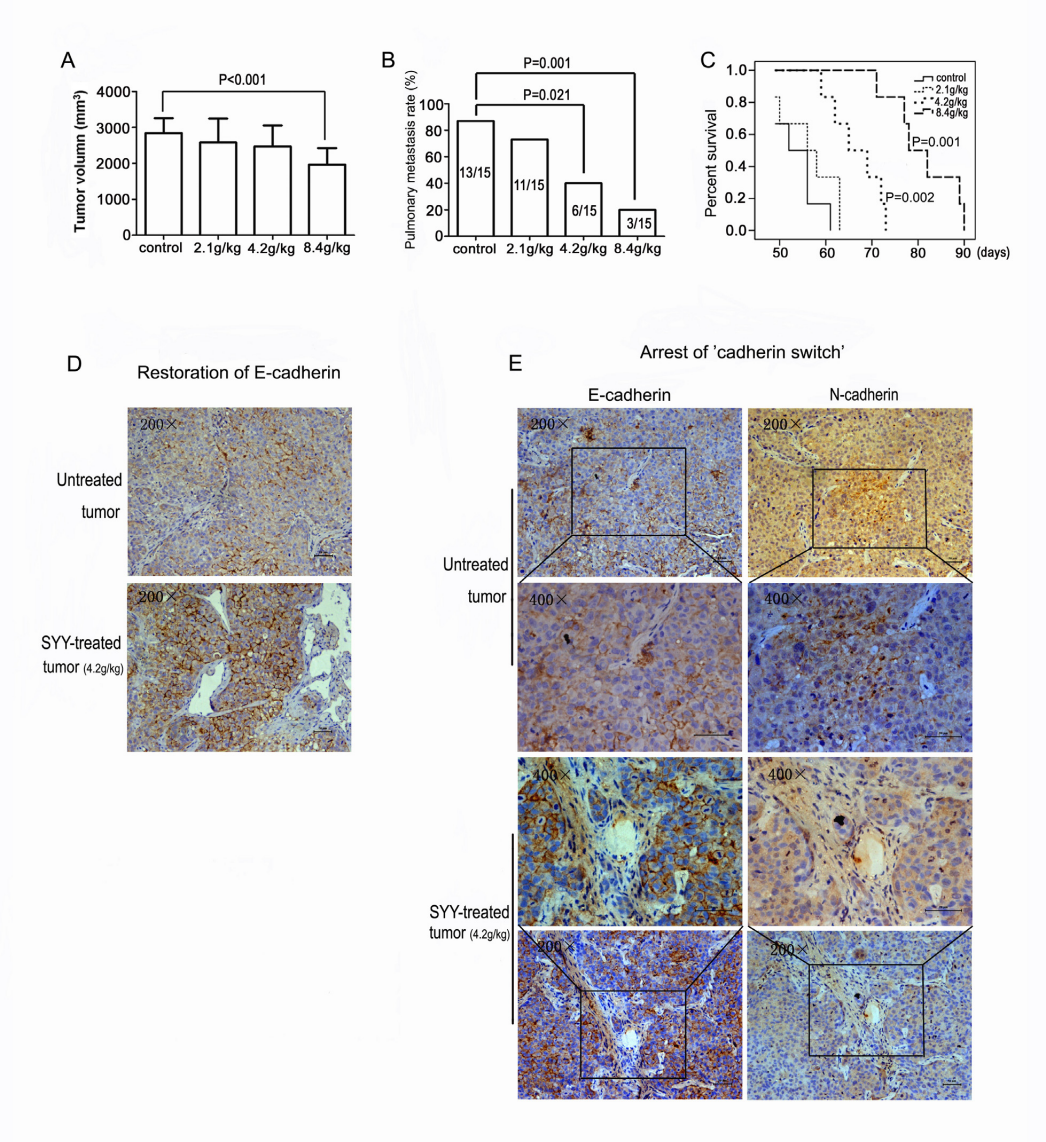
**The effect of Songyou Yin on oxaliplatin pre-treated tumors in nude mice**. Tumor volume was significantly decreased in mice treated with 8.4 g/kg Songyou Yin compared with the control group (A). Songyou Yin treatment (4.2 and 8.4 g/kg) significantly decreased pulmonary metastasis (B) and prolonged survival (C) of nude mice bearing oxaliplatin pre-treated MHCC97L xenografts. Songyou Yin treatment (4.2 g/kg) restored E-cadherin expression (D) and arrested the cadherin switch (E) in tumor tissues.

After 6 weeks, 8.4 g/kg SYY treatment resulted in decreased tumor volume compared with the control group (1969.95 ± 454.32 mm^3 ^vs. 2841.87 ± 417.59 mm^3^; *P *< 0.001). With this SYY dose, maximum inhibition of pulmonary metastasis (3/15 vs. 13/15; *P *= 0.001) and prolonged survival was also observed (81.17 ± 7.36 days vs. 53.83 ± 4.71 days; *P *= 0.001) (Figure 6A-C). In contrast, 2.1 g/kg SYY treatment did not significantly reduce tumor volume, incidence of pulmonary metastasis, or mean survival time. Although 4.2 g/kg SYY did not significantly inhibit tumor growth (2470.41 ± 583.96 mm^3 ^vs. 2,841.87 ± 417.59 mm^3^; *P *= 0.065), this dose decreased the incidence of pulmonary metastasis (6/15 vs. 13/15; *P *= 0.021) and increased life span (66.67 ± 5.61 days vs. 53.83 ± 4.71 days; *P *= 0.002).

To explore the relationship between SYY treatment and EMT, tumor tissues in SYY-treated nude mice and control mice were analyzed by immunohistochemistry. Compared with the control, EMT was less common in tumors from mice treated with 8.4 g/kg or 4.2 g/kg SYY. A restoration of E-cadherin expression (Figure 6D) and a concomitant reduction in N-cadherin expression (Figure 6E) were detected in these tumors. The expression of vimentin was low or even undetectable in most tumors in these two SYY treatment groups.

## Discussion

Chemoresistance and chemotherapy side effects in HCC treatment have been studied; however, little is known about the opposite effect of chemotherapy. Consistent with the studies on other malignant tumors [4-7], we showed that in vitro exposure of MHCC97L and HepG2 HCC cells to pulse treatment with oxaliplatin led to increased motility and invasiveness of the surviving tumor cells. However, in vivo study is more complicated; chemotherapy affects not only tumor cells but also damages blood vessels and inhibits the antitumor system of the host, which may contribute to cancer metastasis [3,8]. Furthermore, chemotherapy directly influences tumor burden, and tumor burden strongly correlates with the incidence of pulmonary metastasis (i.e., metastasis occurs more frequently with larger tumors). To avoid these confounding factors, we employed a re-inoculation model. The similarity in histology between the untreated tumors and oxaliplatin-treated tumors on day 7 after chemotherapy support the validity of this model. Finally, oxaliplatin pre-treated tumors showed a 2.3-fold increase in the incidence of spontaneous metastasis to the lungs of the new recipient mice. These results indicated that HCC cells surviving in vitro and in vivo oxaliplatin treatment have increased metastatic potential, a possible opposite effect of oxaliplatin treatment.

Accumulating evidence indicates that EMT plays an important role in chemotherapy-induced invasiveness in colorectal, pancreatic, breast and ovarian carcinoma cells in vitro. We showed here that pulse exposure to oxaliplatin stimulated transformation of HepG2 and MHCC97L HCC cells from a typical epithelial phenotype to a spindle-shaped mesenchymal phenotype, accompanied by the loss of E-cadherin and upregulation of N-cadherin and vimentin. Further evidence was provided by xenografts in nude mice; molecular alterations consistent with EMT were detected in tumors on day 7 after chemotherapy. These observations were consistent with a recent clinical investigation of breast cancer [21], which indicated that residual breast cancers after chemotherapy and endocrine therapy displayed mesenchymal features. We also detected upregulated transcription factor Snail in oxaliplatin-treated tumor tissues and cell lines. This finding, combined with results of a previous study using oxaliplatin [4], suggests that the Snail signal transduction pathway may play a central role in oxaliplatin-induced tumor progression. Finally, the decreased proliferation of oxaliplatin-treated tumor cells suggests that these tumor cells may have switched from a proliferative to a more invasive and migratory phenotype, thus providing further evidence of EMT following chemotherapy [22].

Interestingly, similar changes were also detected in re-inoculated tumors 6 weeks after re-inoculation. Compared with controls, oxaliplatin pre-treated tumors showed decreased growth and significantly increased EMT. This observation indicates that tumor cells surviving in vivo chemotherapy acquired intrinsic characteristics that facilitated EMT. A possible explanation for this might be derived from the survival mechanisms of tumor cells. Molecules such as interleukin (IL)-8 and integrins not only help tumor cells survive chemotherapy but stimulate EMT [6,23]. These factors may be pre-existing in minor subpopulations of tumor cells and then selected by chemotherapy, or they could be induced by chemotherapy, or both mechanisms may occur. These results, together with enhanced pulmonary metastasis rates in re-inoculated mice, highlight the close relationship among oxaliplatin treatment, EMT, and metastasis. Further investigations are needed to characterize the underlying mechanisms in more detail.

It is not clear whether the increased metastatic potential of residual HCC after oxaliplatin treatment overweighs or is less important to the host than the reduced tumor burden by chemotherapy; however, the opposite effect of chemotherapy attenuates its anti-tumor effect as far as host life span is concerned. As mentioned above, previous clinical trials failed to show a correlation between the HCC patients' response to chemotherapy and prolonged survival. The findings of the present study suggest that the increased metastatic potential within residual HCC cells after chemotherapy may be partly responsible for these results. Indeed, most cancer deaths are due to metastasis in clinical trials [24]. While much attention has been paid to reducing tumor burden, the complex mechanisms involved in chemotherapy-related tumor progression require clarification, which may eventually lead to mechanism-based combination treatments to improve the efficacy of chemotherapy in HCC.

SYY is a Chinese herbal extract consisting of *Salvia miltiorrhiza *Bge. and four other herbs. Some components of SYY have demonstrated value in treating malignancies [25,26]. In our previous study [17], SYY effectively inhibited tumor growth and metastasis, and increased survival in a similar HCC nude mouse model bearing MHCC97H (a cell line with high metastatic potential originating from MHCC97 cells [13]) xenograft. The induction of apoptosis and downregulation of MMP-2 and VEGF were suggested to be responsible for the anticancer effect of SYY. In the present study, we further evaluated the effects of SYY on oxaliplatin pre-treated tumors to determine its effects against chemotherapy-related cancer metastasis. We demonstrated that SYY treatment at doses of 4.2 g/kg and 8.4 g/kg significantly inhibited the metastatic potential of residual HCC after chemotherapy and prolonged mouse survival. Thus SYY treatment may attenuate the opposite effect of oxaliplatin treatment, thereby improving treatment efficacy. Furthermore, the restoration of E-cadherin and arrest of cadherin switch in SYY-treated tumors suggest that EMT inhibition may play a role in SYY effects. In addition to recent studies that demonstrated EMT attenuation by a herbal prescription Wen-pi-tang-Hab-Wu-ling-san and a medicinal herb Panax notoginseng in kidney cells against fibrosis[27,28], we showed here that the arrest of EMT was also linked to the anticancer effects of traditional Chinese medicine. Expression of MMP2 and VEGF was found to be important in the EMT program, with relevance to the induction of transcription factor Snail [21,23,29]. Hence, the attenuation of EMT by SYY demonstrated in the present study supports our previous findings by providing underlying mechanisms. One limitation of the present study is the lack of detailed understanding of the relationship between EMT arrest and SYY treatment due to its complexity. Therefore, additional basic research is needed.

## Conclusions

Our results suggest that residual HCC cells after oxaliplatin treatment undergo EMT and exhibit increased metastatic potential. Accordingly, attenuation of EMT by SYY may improve the efficacy of chemotherapy in HCC.

## Competing interests

The authors declare that they have no competing interests.

## Authors' contributions

WX, ZYT, ZGR, HCS, SJQ, LW, BBL and QSL contributed to the study design, analysis and interpretation of data. ZYT conceived the study. WX performed the experiment. WZ, WQW and LL participated in the establishment of nude mice model. XDZ participated in statistical analysis. WX and ZGR drafted the manuscript. ZYT carried out the revision and provided important suggestions. All authors approved the final manuscript.

## Pre-publication history

The pre-publication history for this paper can be accessed here:

http://www.biomedcentral.com/1471-2407/10/219/prepub

## Supplementary Material

Additional file 1 **The fingerprints of Songyou Yin and its five characteristic components**. Fingerprinting of Songyou Yin and its five characteristic components was performed by high-performance liquid chromatography (HPLC) (A for Songyou Yin; B1 for *Salvia miltiorrhiza *Bge.; B2 for *Astragalus membranaceus *Bge.; B3 for *Lycium barbarum *L.; B4 for *Crataegus pinnatifida *Bge. and B5 for *Trionyx sinensis *Wiegmann). Click here for file

Additional file 2 **Expression of Ki-67 in untreated and oxaliplatin pre-treated tumors**. Immunohistochemistry showed a significant reduction of Ki-67 positive cells in oxaliplatin pre-treated tumors. Click here for file

Additional file 3 **Pulmonary metastatic foci in nude mouse bearing oxaliplatin pre-treated MHCC97L xenograft**. Click here for file

Additional file 4 **Oxaliplatin pre-treated tumors exhibited changes consistent with epithelial-mesenchymal transition (EMT) in re-inoculated nude mice**. Immunohistochemistry showed a decreased expression of E-cadherin with an upregulation of N-cadherin, vimentin and Snail in oxaliplatin pre-treated tumors compared with untreated tumors. Click here for file
