# Supplementary figures and images for: Residual hepatocellular carcinoma after oxaliplatin treatment has increased metastatic potential in a nude mouse model and is attenuated by Songyou Yin
Source: BMC Cancer. 2010 May 20;10:219. doi: 10.1186/1471-2407-10-219 (PMC2880993; doi:10.1186/1471-2407-10-219)

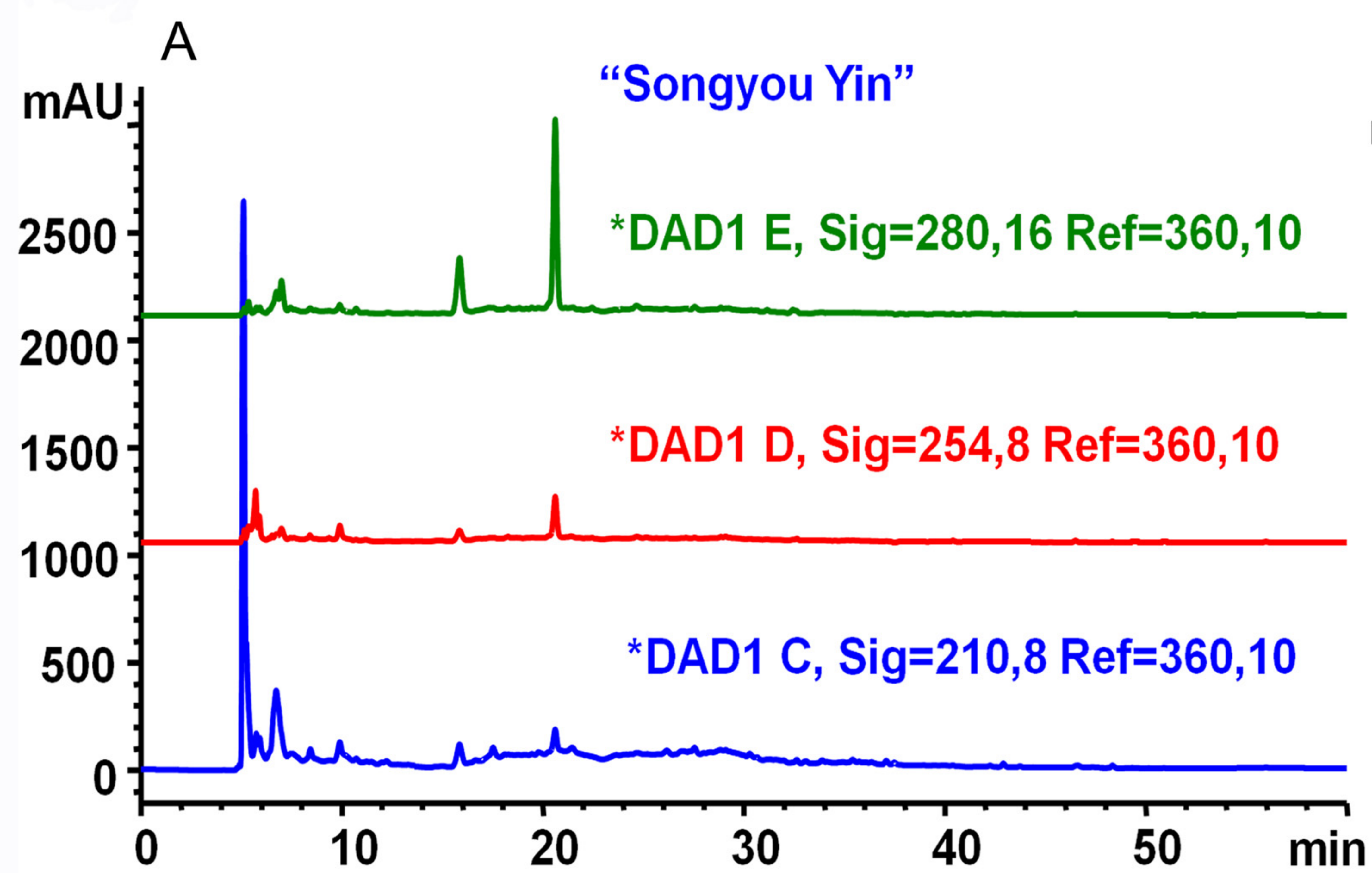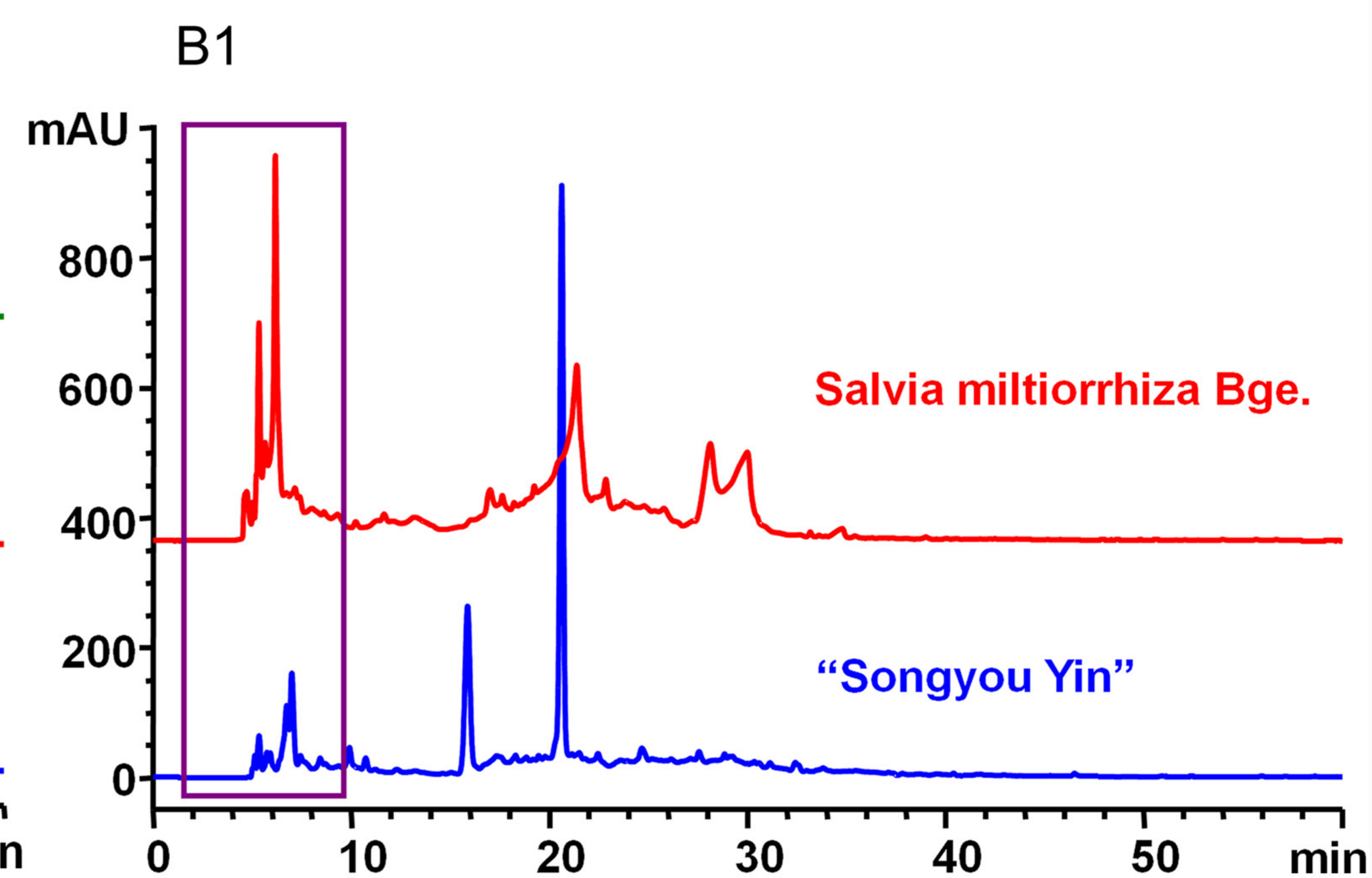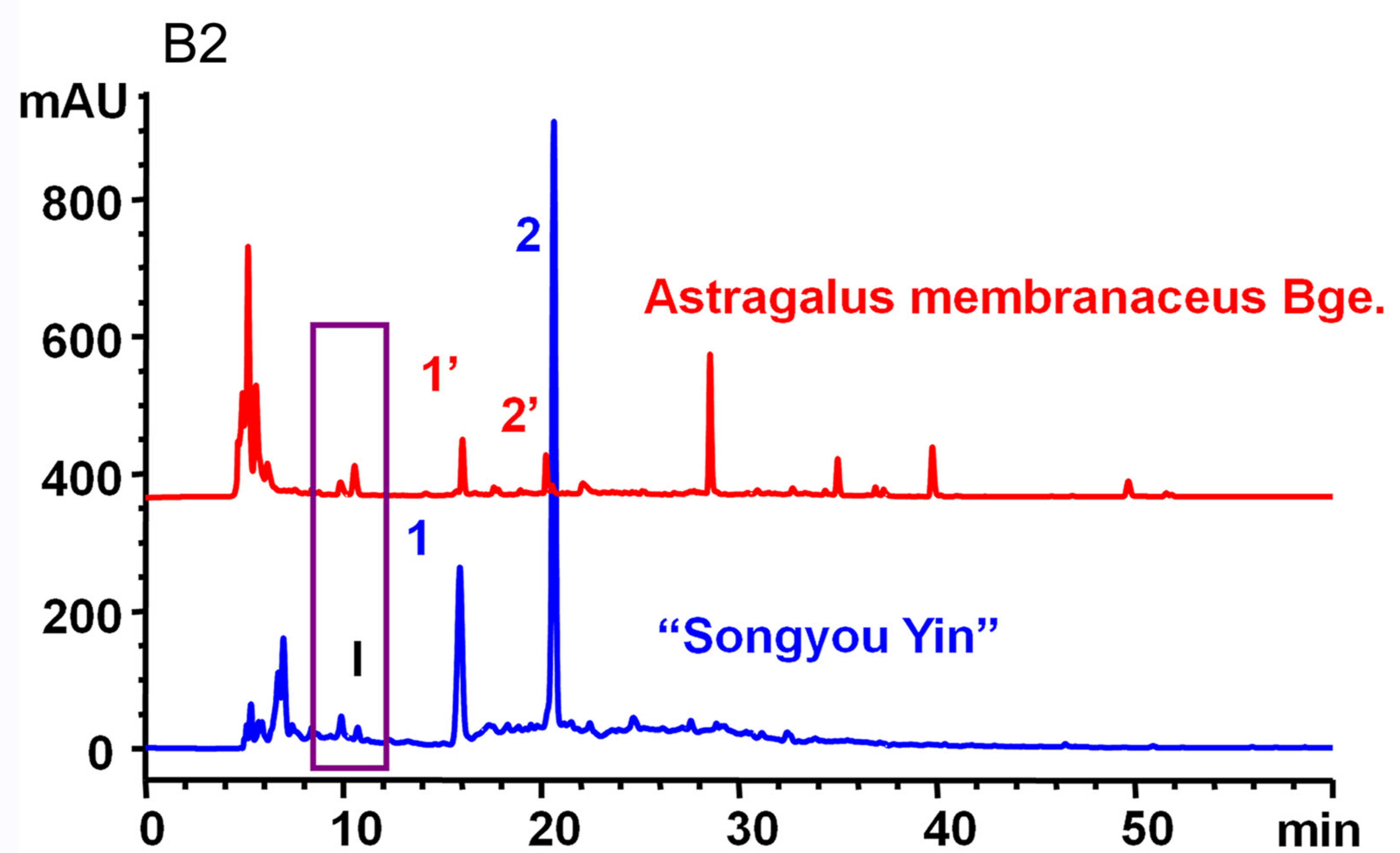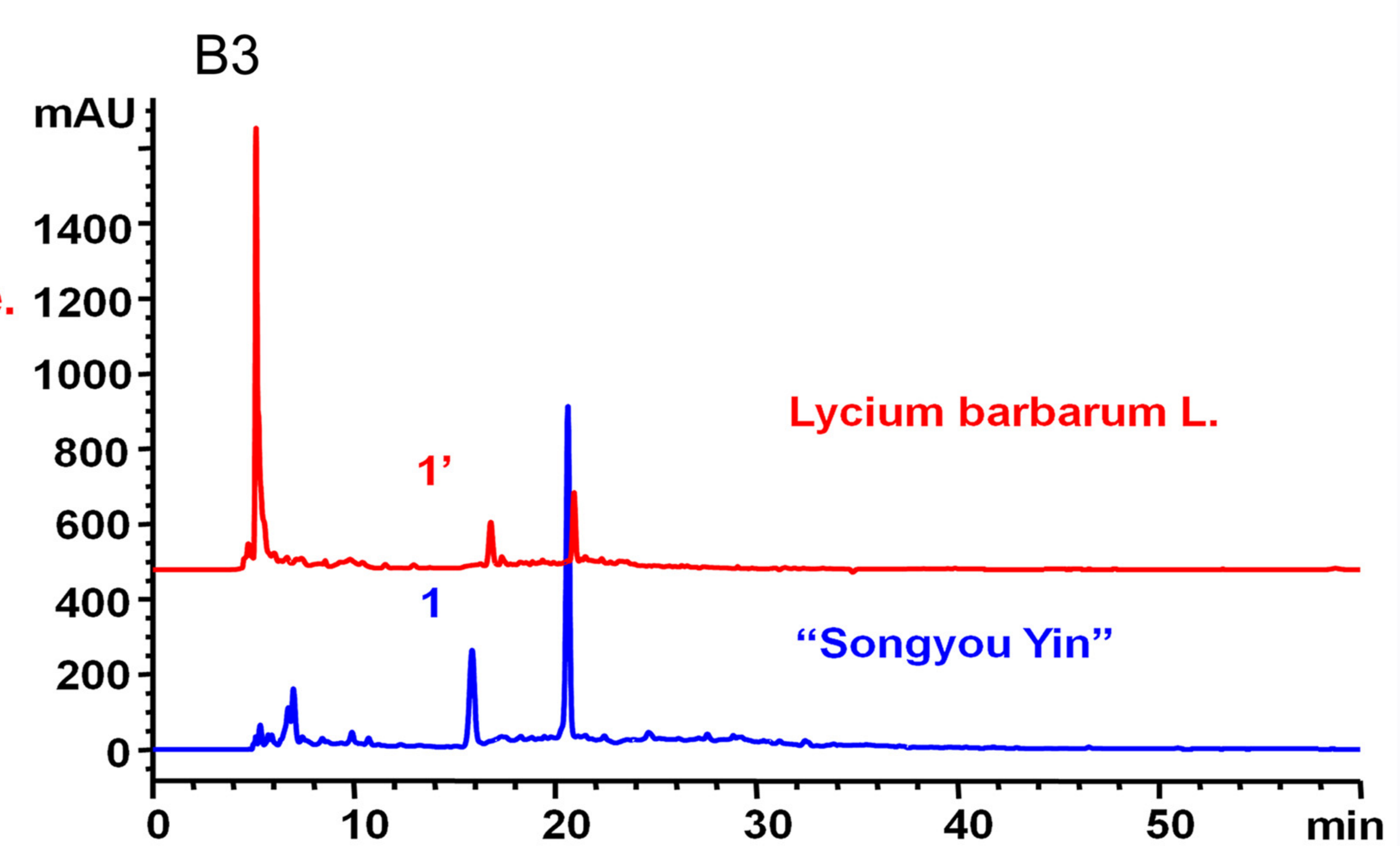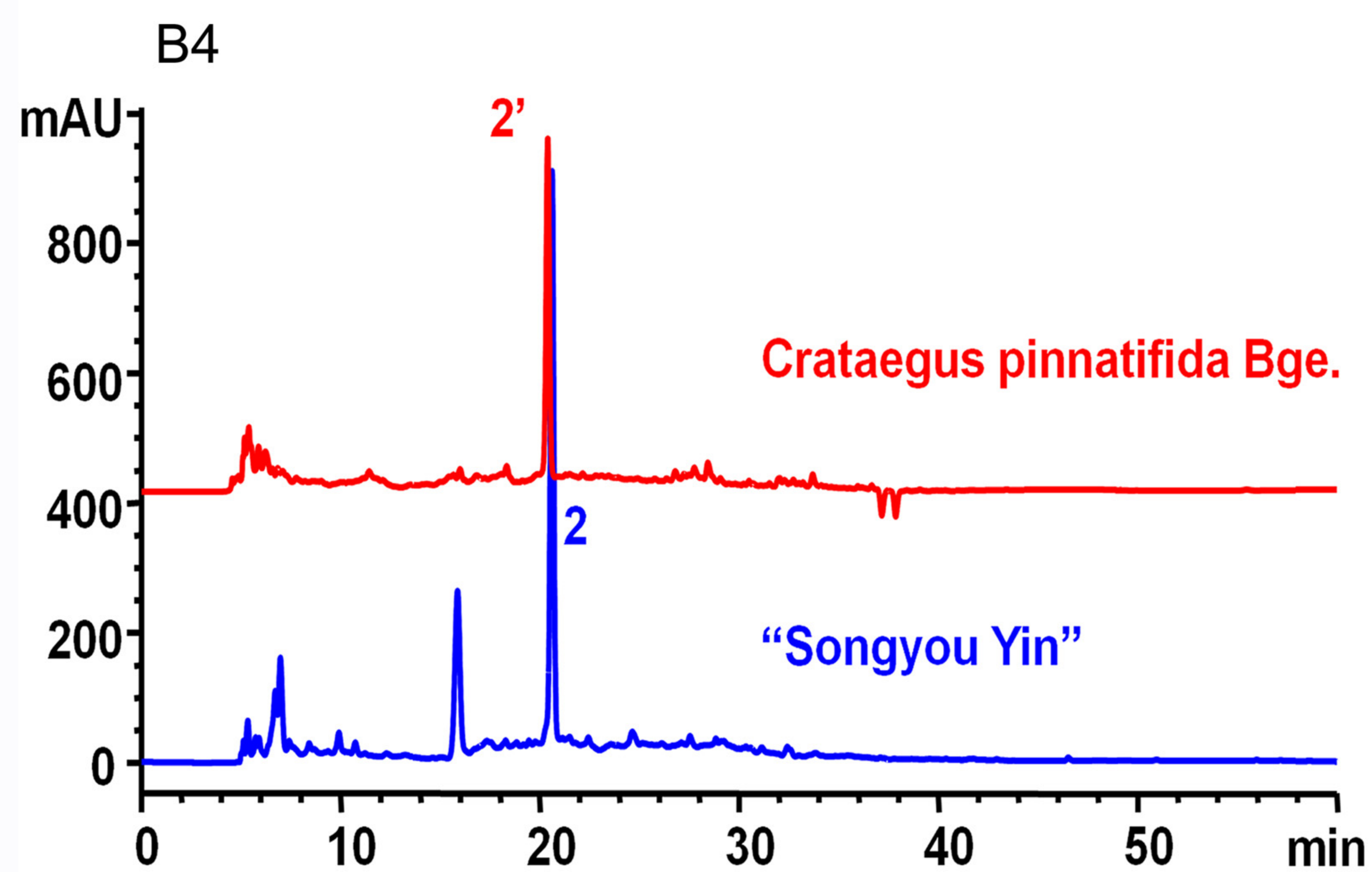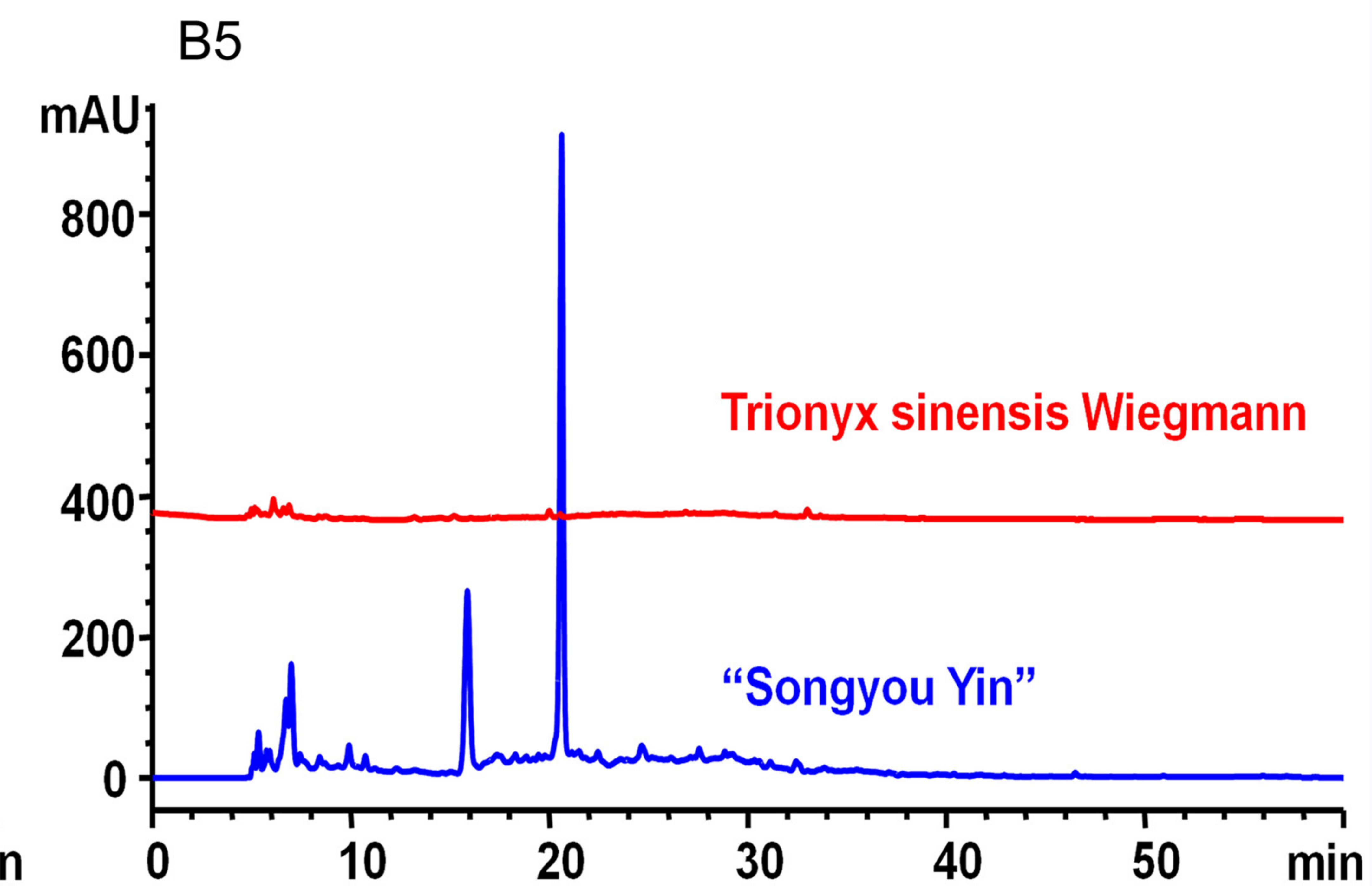

Supplement: Additional file 1 — The fingerprints of Songyou Yin and its five characteristic components. Fingerprinting of Songyou Yin and its five characteristic components was performed by high-performance liquid chromatography (HPLC) (A for Songyou Yin; B1 for Salvia miltiorrhiza Bge.; B2 for Astragalus membranaceus Bge.; B3 for Lycium barbarum L.; B4 for Crataegus pinnatifida Bge. and B5 for Trionyx sinensis Wiegmann). [file 1471-2407-10-219-S1.PDF]

# Ki-67

Untreated  
tumor

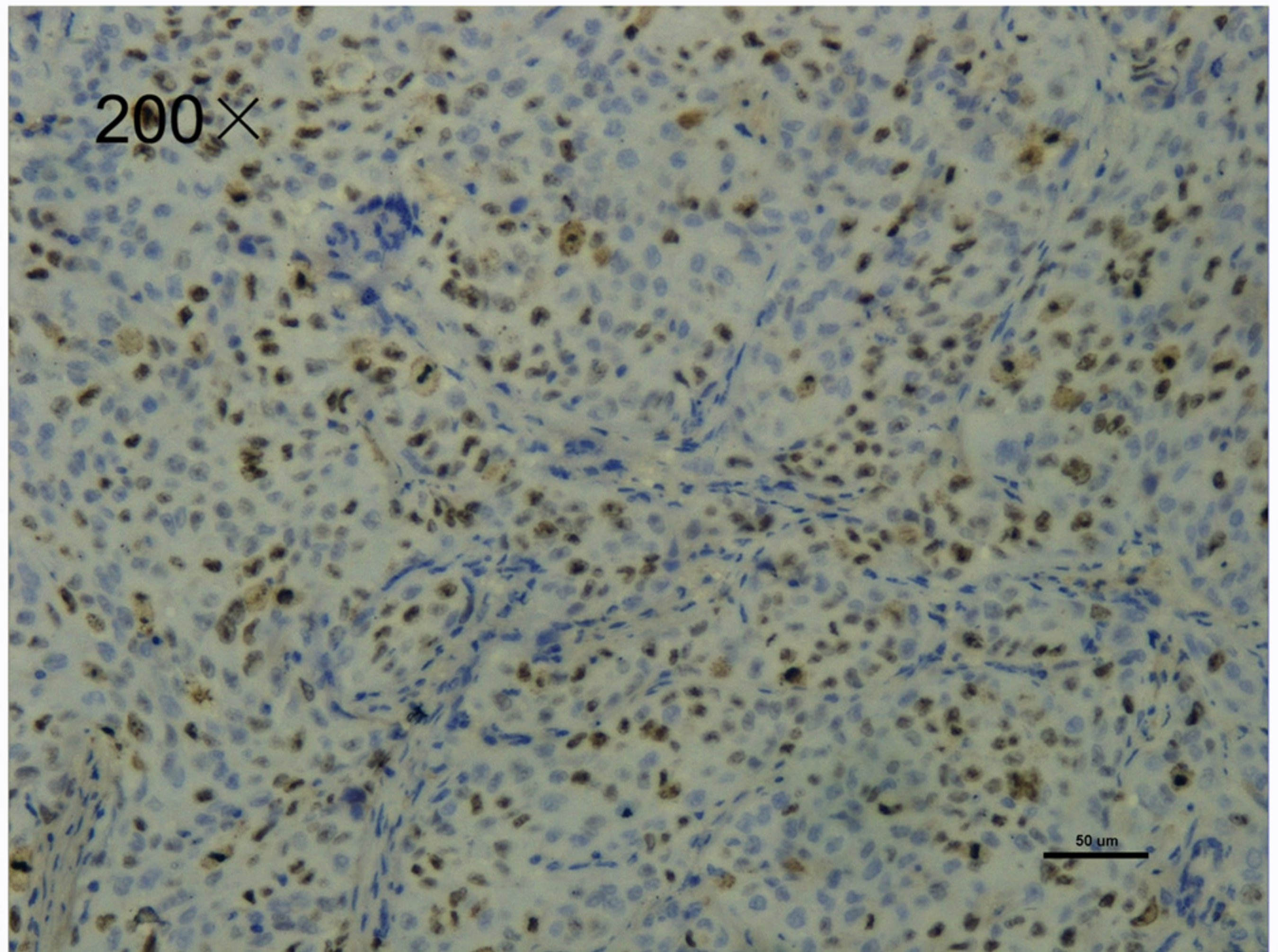

Oxaliplatin  
pre-treated  
tumor

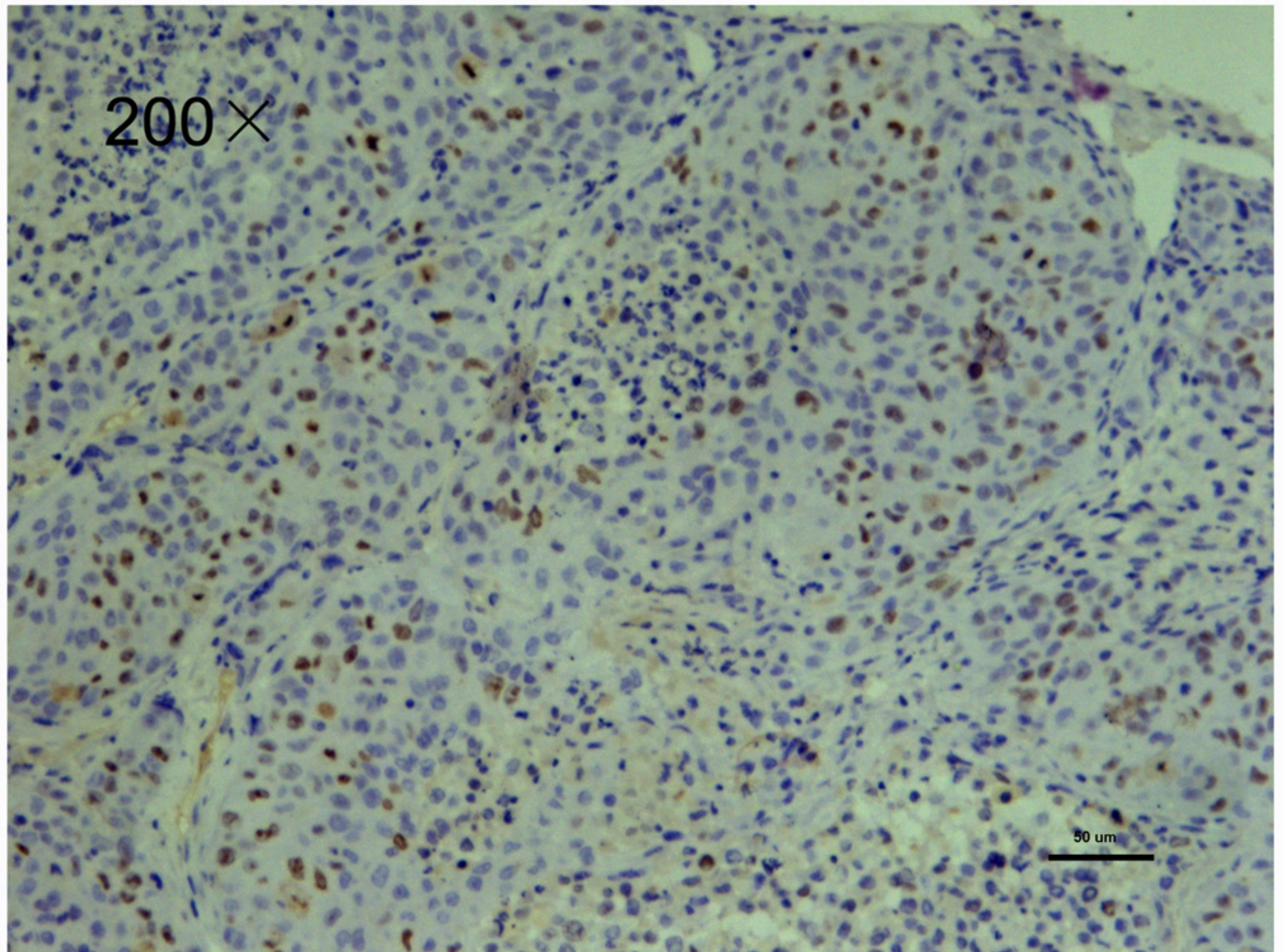

Supplement: Additional file 2 — Expression of Ki-67 in untreated and oxaliplatin pre-treated tumors. Immunohistochemistry showed a significant reduction of Ki-67 positive cells in oxaliplatin pre-treated tumors. [file 1471-2407-10-219-S2.PDF]

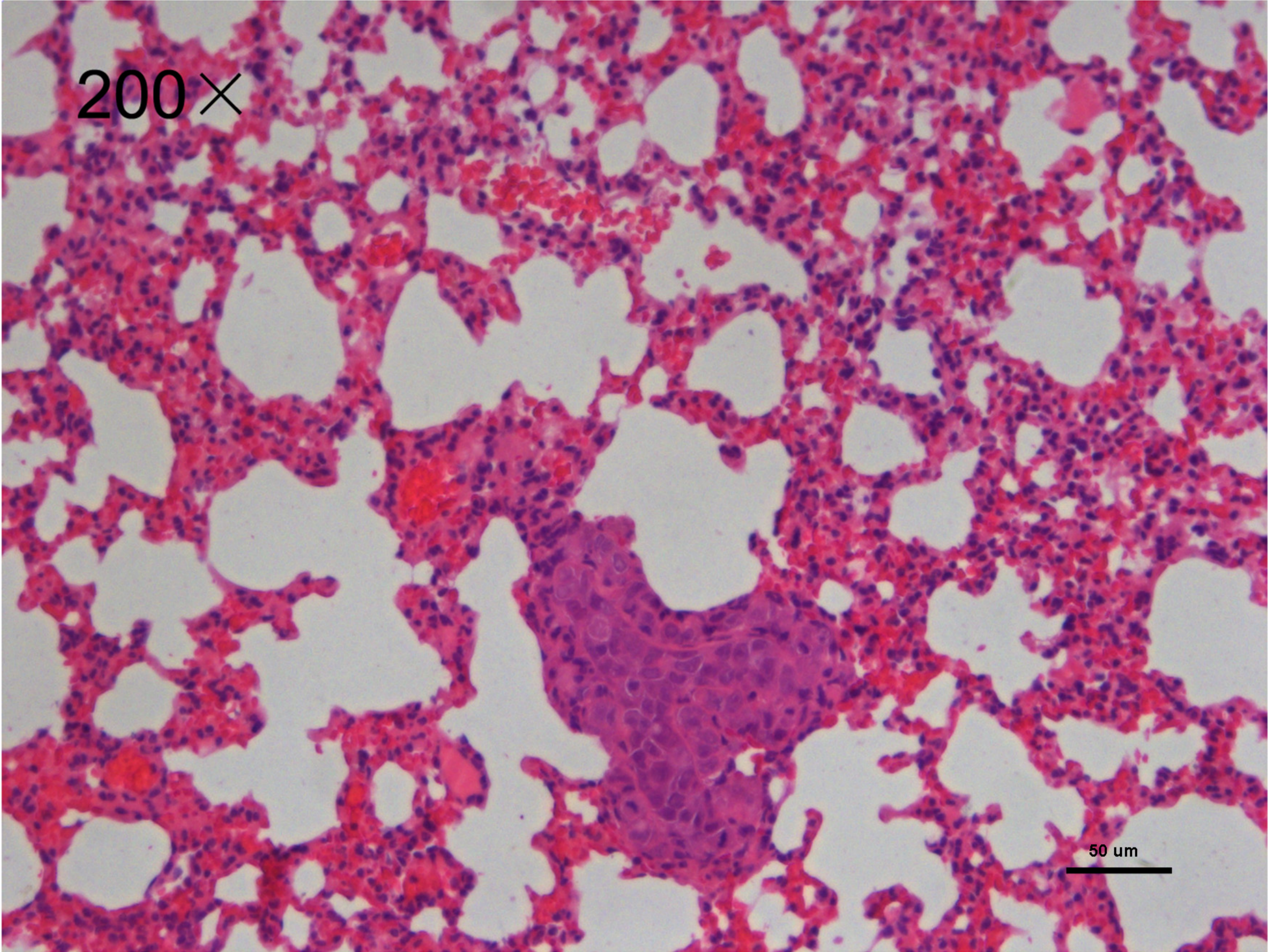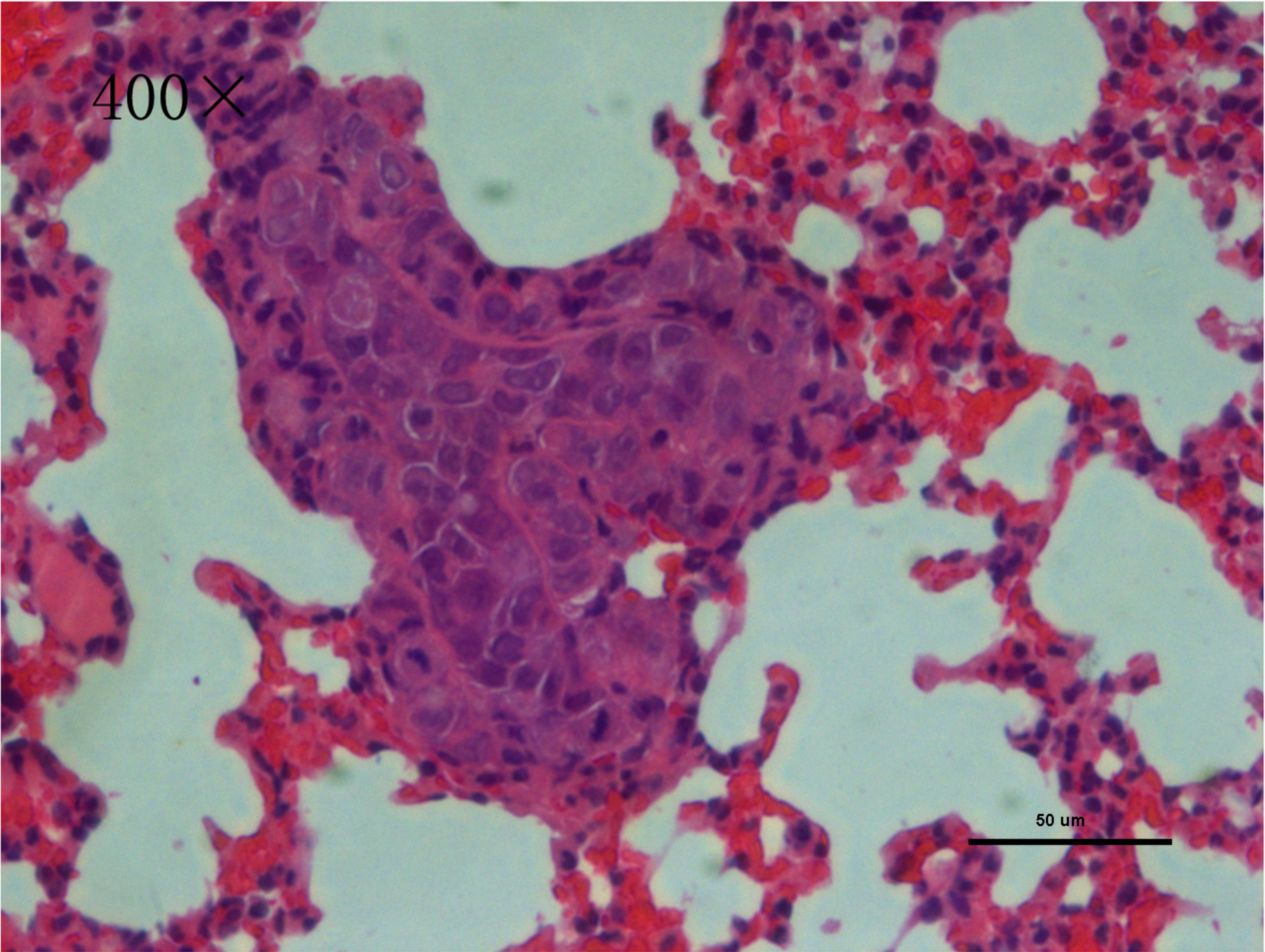

Supplement: Additional file 3 — Pulmonary metastatic foci in nude mouse bearing oxaliplatin pre-treated MHCC97L xenograft. [file 1471-2407-10-219-S3.PDF]

E-cadherin

N-cadherin

Vimentin

Snail

Untreated  
tumor

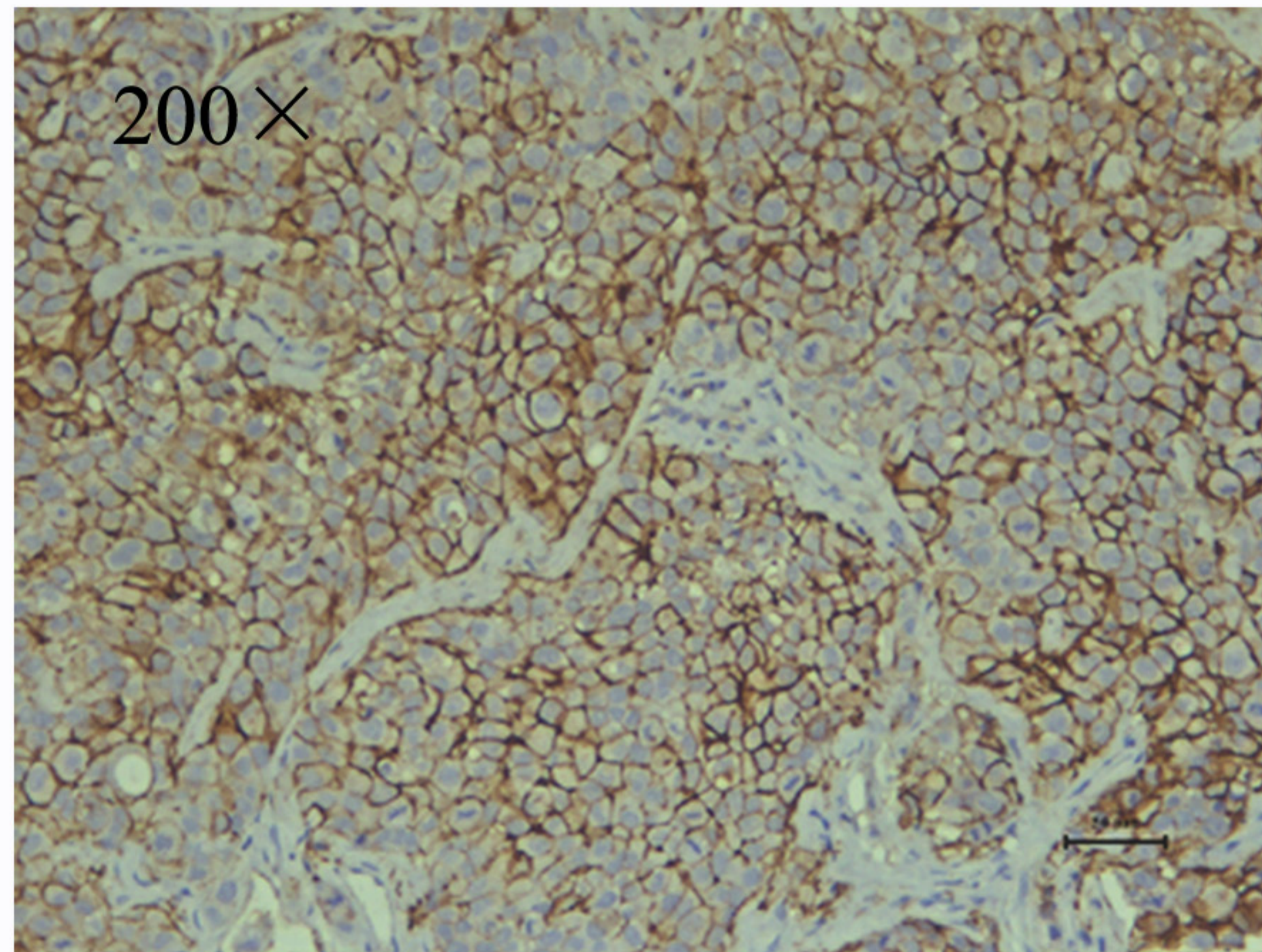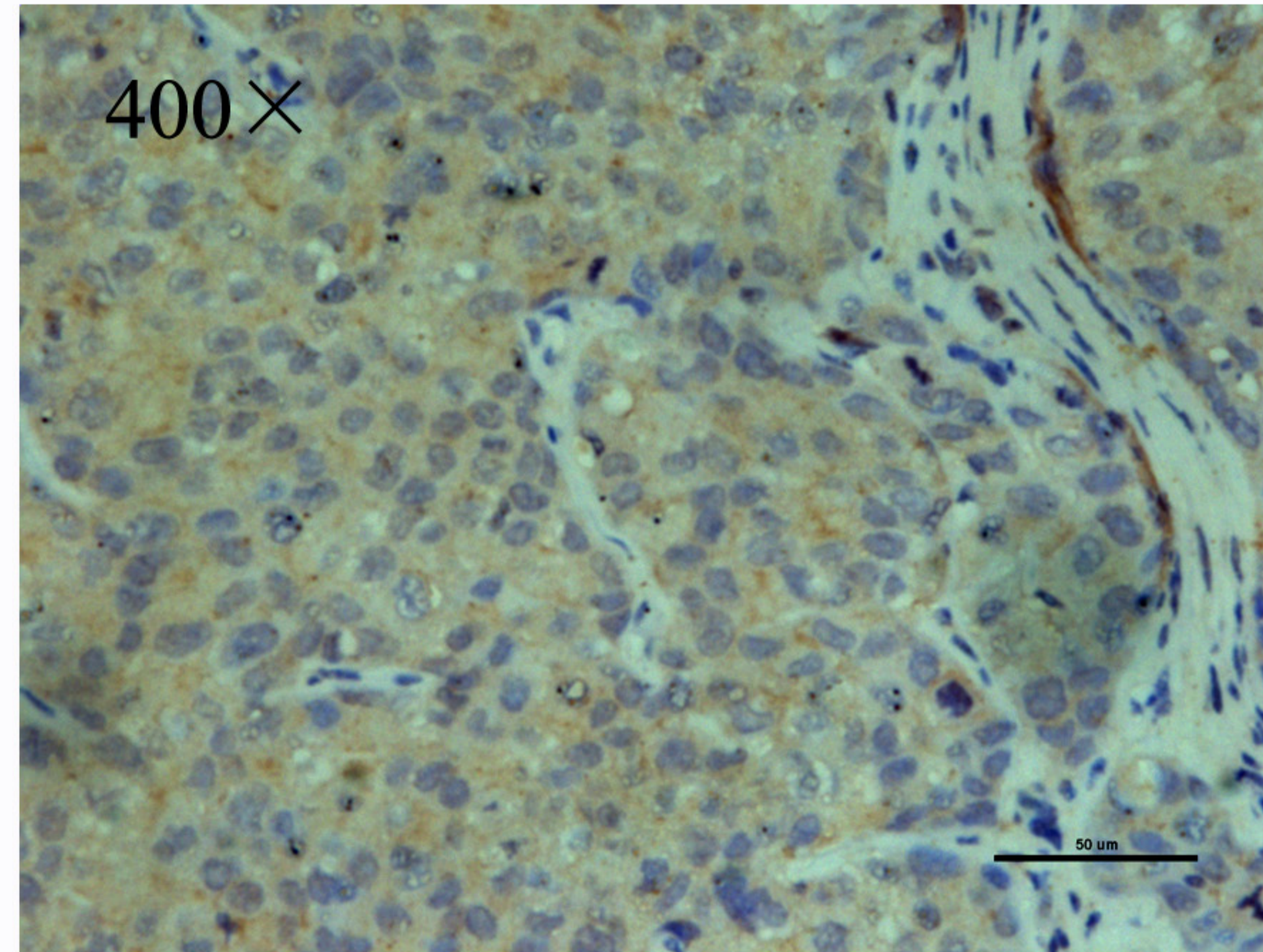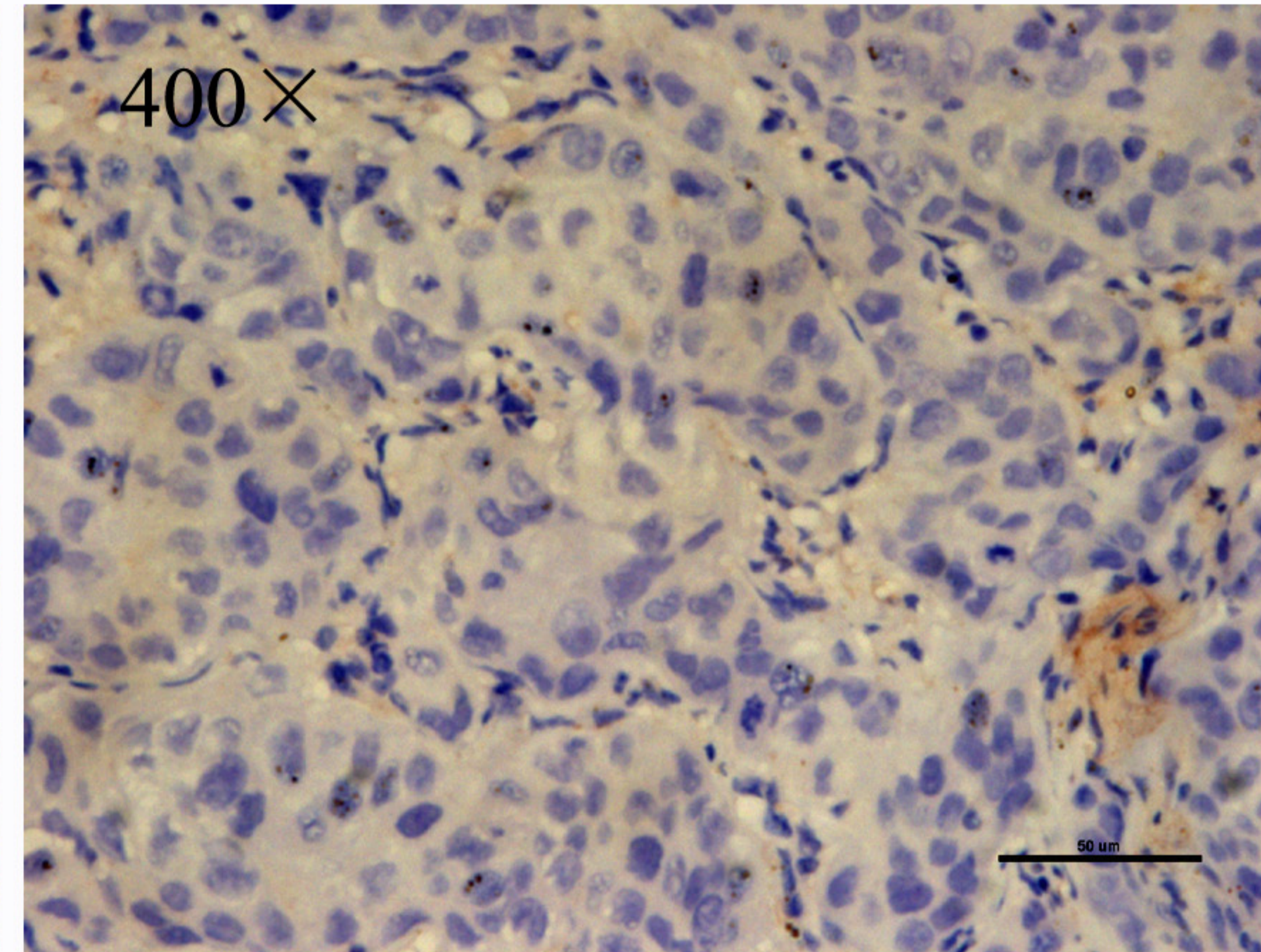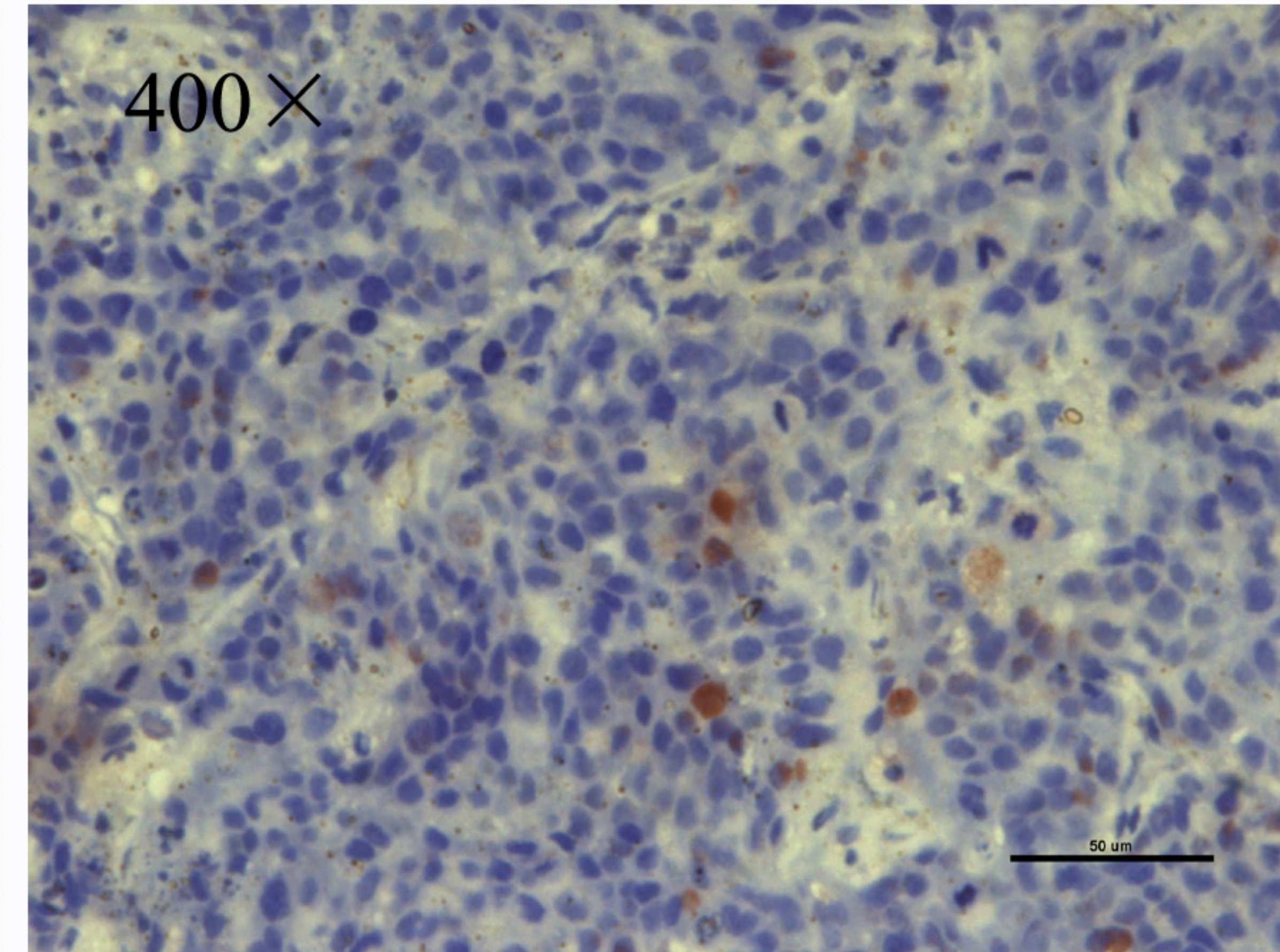

Oxaliplatin  
pre-treated tumor

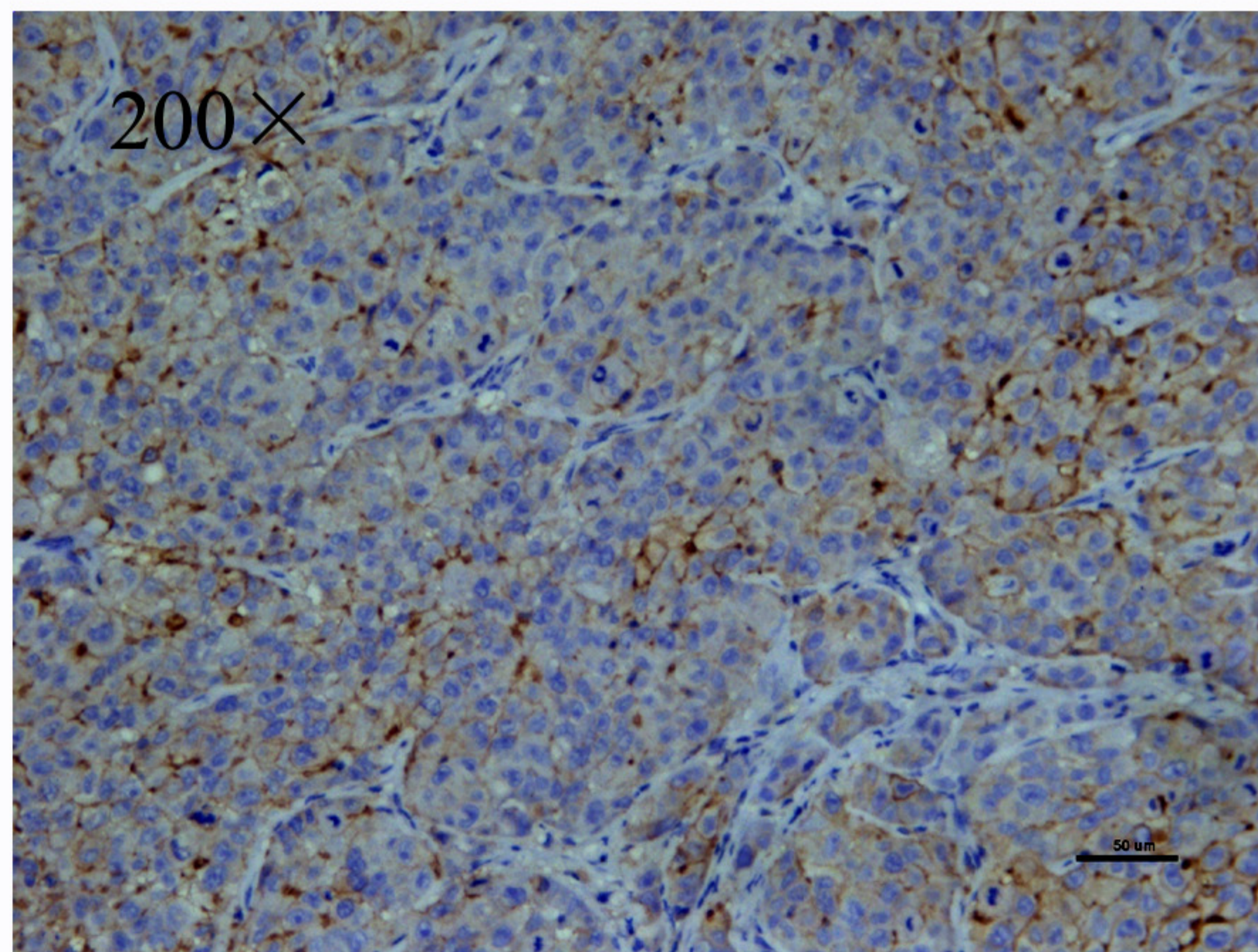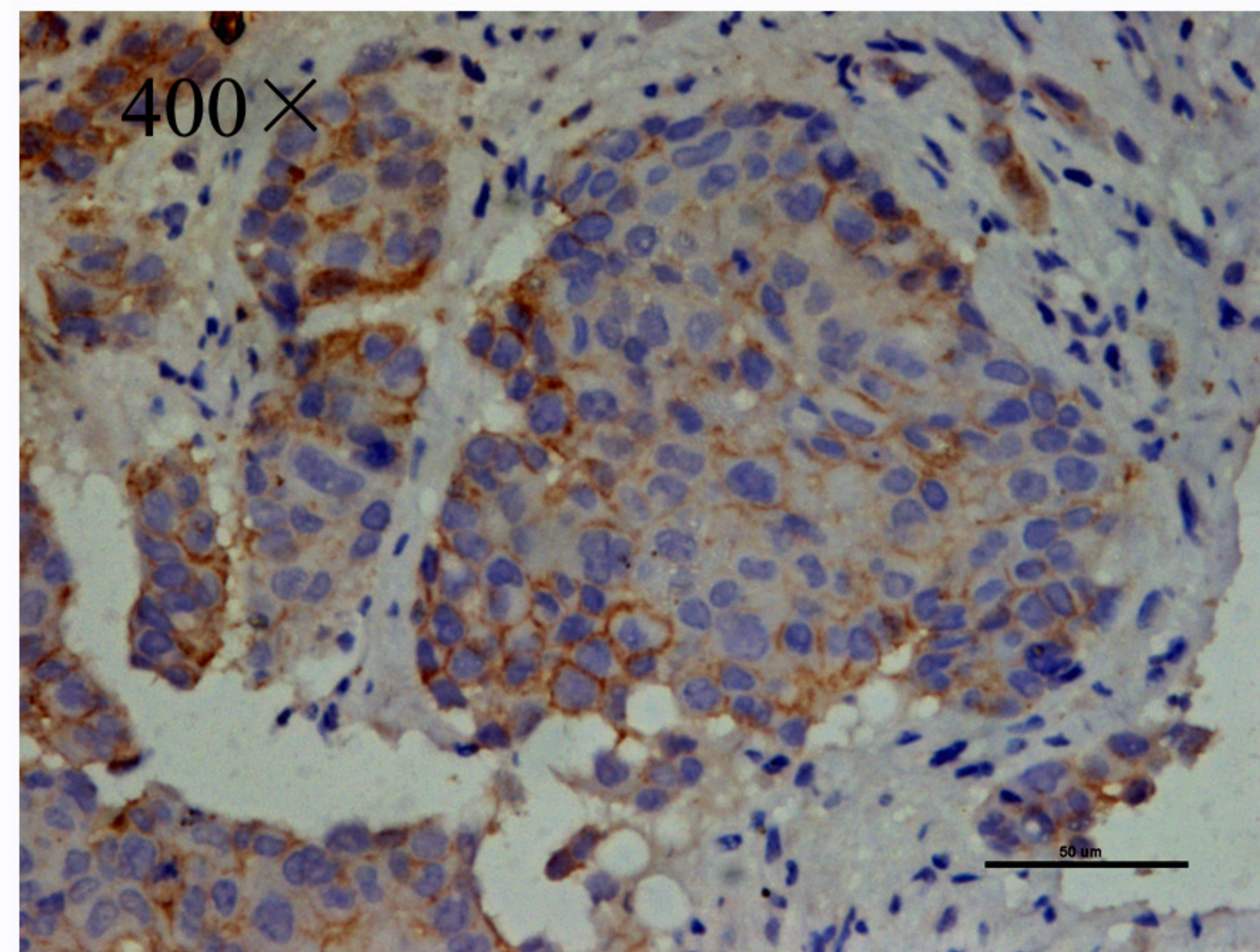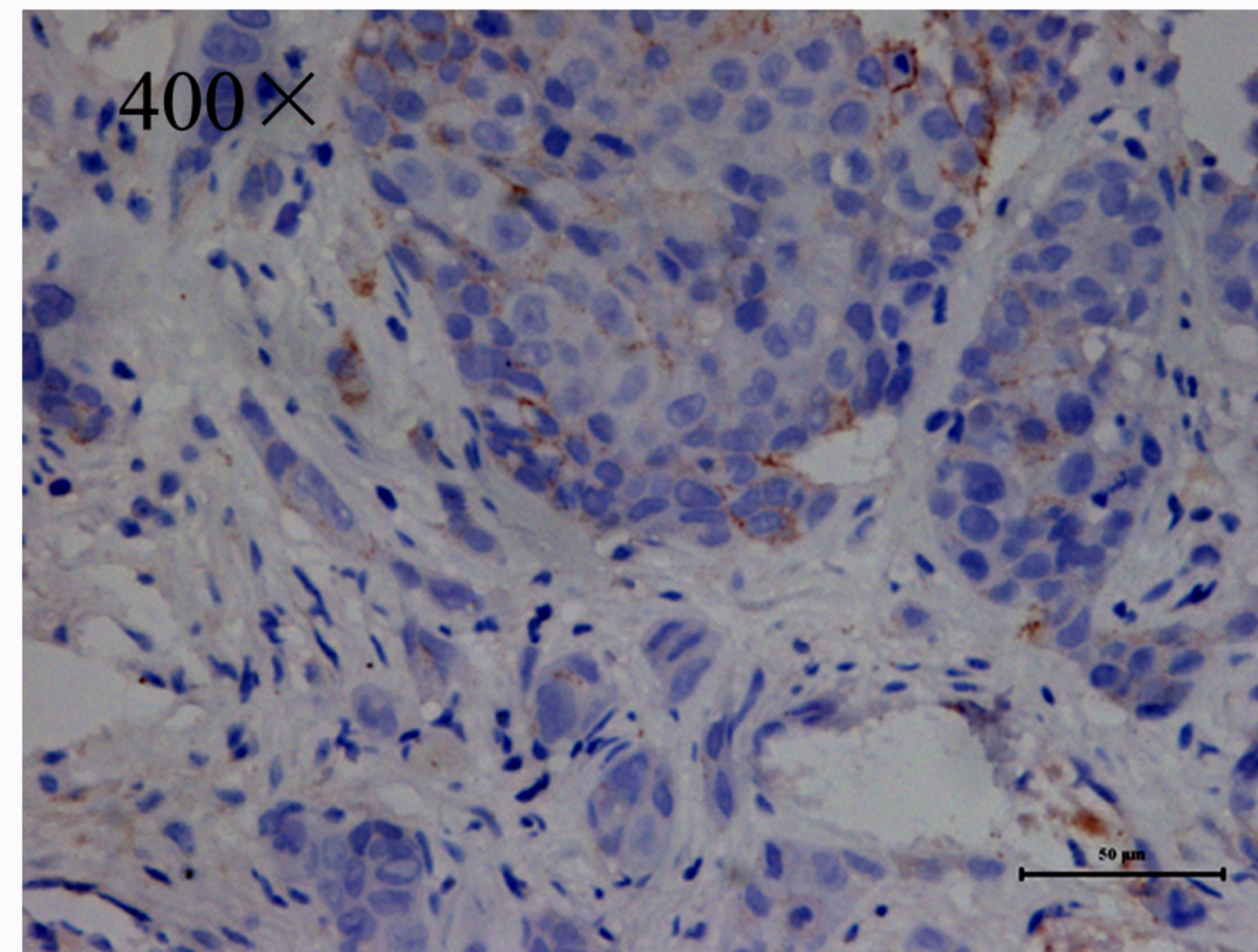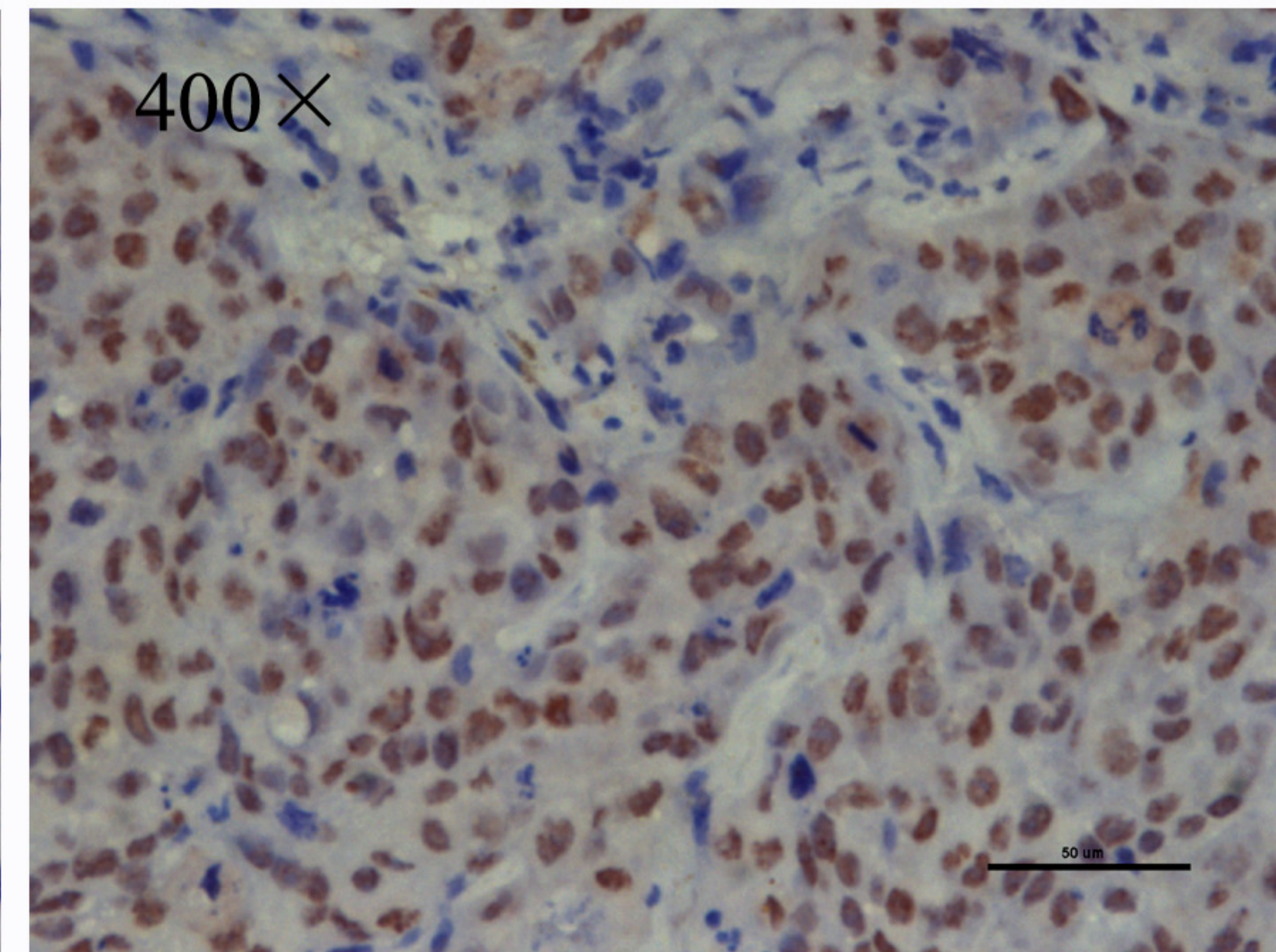

Supplement: Additional file 4 — Oxaliplatin pre-treated tumors exhibited changes consistent with epithelial-mesenchymal transition (EMT) in re-inoculated nude mice. Immunohistochemistry showed a decreased expression of E-cadherin with an upregulation of N-cadherin, vimentin and Snail in oxaliplatin pre-treated tumors compared with untreated tumors. [file 1471-2407-10-219-S4.PDF]
